# *Astragalus mongholicus* *bunge* and *Angelica sinensis* botanical drug decoction mitigates lung inflammation through NOX4/TGF-β1/SMAD3 signaling

**DOI:** 10.3389/fphar.2025.1565569

**Published:** 2025-03-26

**Authors:** Zhifeng Yang, Yuqian Chang, Tong Zhou, Wenwen Sui, Ping Dai, Yuan Wei, Jia-Sheng Wang, Jun Zhou, Chengli Wen, Haidong Zhang

**Affiliations:** ^1^ Shandong Academy of Occupational Health and Occupational Medicine, Shandong First Medical University and Shandong Academy of Medical Sciences, Jinan, Shandong, China; ^2^ Affiliated Hospital of Shandong University of Traditional Chinese Medicine, Jinan, Shandong, China; ^3^ People’s Hospital of Dongying District, Dongying, Shandong, China; ^4^ Interdisciplinary Toxicology Program and Department of Environmental Health Science, College of Public Health, University of Georgia, Athens, GA, United States; ^5^ Shandong Center for Disease Control and Prevention, Jinan, Shandong, China

**Keywords:** pulmonary fibrosis, lung inflammaiton, botanical drug decoction, NOX4, Danggui Buxue Tang

## Abstract

**Introduction:**

*Astragalus mongholicus bunge* and *Angelica sinensis* are botanical drugs rich in beneficial nutrients and health-promoting metabolites. Their roots can be decocted to a botanical drug decoction “Danggui Buxue Tang (DBT),” demonstrating human anti-inflammatory

**Methods:**

Here, we evaluate the mitigating function of DBT on lung inflammation and early fibrosis in a rat model. The model was established by tracheal dripping of silica suspension for 28 days. Positive intervention effects of DBT were observed in a dose-dependent manner after consecutive gavage of DBT (1.9, 3.8, and 7.6 g/kg·bw/d) for 28 days and 42 days. To explore the underlying molecular mechanism. DBT metabolites were profiled using a liquid chromatograph-mass spectrometer and the Chemspider database.

**Results:**

Lung inflammation and fibrosis were confirmed using functional tests and histopathologic analysis. Metabolite target analysis identified nicotinamide adenine dinucleotide phosphate (NADPH) oxidase 4 (NOX4) as a key target of DBT in regulating pulmonary fibrosis. Gene ontology (GO) analysis estimated that oxidative stress, inflammatory response, myofibroblast differentiation, and extracellular matrix (ECM) deposition were the major target pathways of DBT. KEGG analysis found that DBT might modulate pulmonary fibrosis through the transforming growth factor-β (TGF-β) pathway. GO chord and signaling pathway maps revealed that NOX4 contributes to oxidative stress, inflammatory response, and TGF-β pathway regulation. The *in vivo* analyses confirmed that DBT significantly reduces NOX4 protein expression, inhibits oxidative stress and inflammatory responses, and reduces TGF-β1, p-SMAD3, fibronectin 1 (FN1), and smooth muscle actin (α-SMA) protein expression.

**Discussion:**

These findings demonstrate the lung-protecting function of DBT in a rat model and identify critical target proteins associated with the underlying mechanism.

## Graphical Abstract

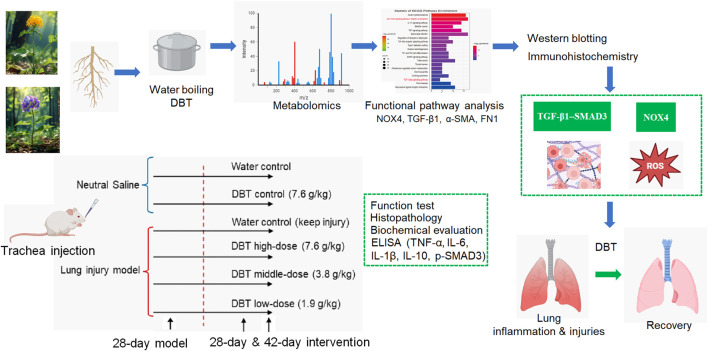

## 1 Introduction

Traditional Chinese medicine (TCM) comprises naturally-derived compounds that have served as medicine and dietary supplements in China for thousands of years (Wang et al., 2025). TCM botanical drug decoctions are a specific combination of two or more botanical drugs prepared by a unique method (Ng et al., 2023).

Numerous pulmonary infectious disease outbreaks have had considerable impacts globally, including the 2003 severe acute respiratory syndrome (SARS) outbreak and the coronavirus disease 2019 (COVID-19) pandemic (Baker et al., 2022). Pulmonary inflammation, early fibrosis, and tissue injury are commonly reported in these diseases and impede patient recovery (Upadhya et al., 2022; Erickson et al., 2023; Rahman et al., 2022). Anti-inflammatory agents are commonly administered to patients; however, they can have adverse effects in specific populations, including children, older individuals, and pregnant people (Sohail et al., 2023). TCM botanical drug decoctions with reported anti-inflammatory and injury-recovery functions (Adamcakova and Mokra, 2021) may provide alternative therapeutic options for these vulnerable patients (Huda et al., 2024). During China’s COVID-19 containment, 92% of confirmed cases were treated with combined TCM and allopathic medicine, achieving over 90% clinical remission or symptomatic improvement (Huang et al., 2021). Moreover, a retrospective cohort study demonstrated that administering *Qingfeipaidu* decoction for 6 days to patients with COVID-19 accelerated symptom (fever and cough) resolution, normalized inflammatory markers (Lymph %, c-reactive protein, and erythrocyte sedimentation rate), and mitigated CT-documented pulmonary inflammation (Xin et al., 2020).

*Astragalus mongholicus bunge* and *Angelica sinensis* are botanical drugs rich in nutrients and metabolites with ameliorative effects on lung inflammation (Nai et al., 2021). Specifically, *A. mongholicus bunge* metabolites exhibit antioxidant, anti-inflammatory, and immunomodulatory effects (Zhu et al., 2022) that can reduce oxidative stress and inhibit the release of inflammatory factors, improving the inflammatory state of the lungs (Zhu et al., 2022). *Angelica sinensis* also exerts protective effects on lung tissue, reducing inflammatory cell infiltration and decreasing the expression of fibrosis-related proteins (Zhi et al., 2024). The roots of the two plants can be decocted to a botanical drug decoction named “Danggui Buxue Tang” (DBT), which has been utilized for centuries in China to replenish blood, enhance immunity, and improve body functions (Lu et al., 2022).

In the theoretical framework of TCM, the pathogenesis of lung injury is attributed to prolonged, uncontrolled, and excessive coughing, leading to lung qi failure and blood stagnation (Huang et al., 2021). Coughing can impede lung qi propagation, causing breathing abnormalities (e.g., severe cough, asthma, etc.), potentially related to airway mucosal damage (Liu et al., 2024). Inhaling toxic substances can cause chronic coughing and persistent mechanical stimulation, resulting in the detachment of respiratory ciliated epithelial cells, impaired mucus–cilia clearance, and increased airway hyper-responsiveness, exacerbating related upper respiratory tract symptoms. Improper clearance can cause qi and blood to run through the lungs, accumulating heat and producing pathological effects, including phlegm, stasis, and qi and blood stagnation. Ding reported that pulmonary infiltration and activation of macrophages and neutrophils correspond to heat toxins obstructing the lungs and stasis in the pulmonary capillaries and interstitium (Ding et al., 2020). In the early stage of lung injury, activated neutrophils and macrophages release pro-inflammatory factors (e.g., tumor necrosis factor (TNF)-α and interleukin (IL)-1β), damaging vascular endothelium and leading to lung congestion.

DBT is commonly used to treat lung injury and early pulmonary fibrosis by tonifying lung qi, activating blood circulation, and removing blood stasis. Mechanistically, astragaloside IV, the main DBT metabolite, increases mitochondrial biosynthesis, improves alveolar type II epithelial cell energy metabolism, and promotes alveolar epithelial barrier repair, thus “tonifying the lungs and benefiting the qi” (Yuan et al., 2023). Moreover, DBT metabolite ferulic acid can inhibit inflammatory factors induced by exudation and tissue congestion, exerting a “blood circulation and eliminate blood stasis” effect (Han et al., 2021). Meanwhile, the total glucosides extracted from *A. mongholicus bunge* and *A. sinensis* can ameliorate bleomycin (BLM)-induced pulmonary fibrosis in a rat model (Zhao et al., 2015). However, it remains unclear whether whole DBT, rather than its purified metabolites, can also alleviate or promote recovery from lung inflammation, early fibrosis, and tissue injury.

Considering that water extraction effectively preserves the bioactivity of polar metabolites (Lin et al., 2017), in this study, we evaluate the capacity of aqueous DBT decoctions to mitigate lung inflammation and early fibrosis in a silica exposure rat model. Based on non-targeted metabolomics and functional pathway analysis, we perform a predictive functional analysis of DBT. We also validate the function and target proteins in a rat model. The findings of this study advance the knowledge of the lung-protecting effects of DBT as a simply prepared TCM botanical drug decoction.

## 2 Methods and material

### 2.1 Animal study

Sixty male Wistar rats (170–200 g, 6–8-week-old) were provided by Jinan Pengyue Laboratory Animal Breeding Co., Ltd. (Laboratory Animal Production License No. SCXK (Lu) 20220006, Jinan, China). The rats were housed in a specific pathogen-free (SPF) rank facility at the Shandong Provincial Centre for Disease Control and Prevention, with unrestricted access to water and food, at 23°C ± 2°C and 40%–70% relative humidity. Throughout the study, a pain management program was implemented to minimize animal discomfort. All animal experiments were performed strictly with the Laboratory Animal Guideline for Ethical Review of Animal Welfare (GB/T 35892-2018, China). The animal study was reviewed and approved by the Ethics Committee of Shandong Academy of Occupational Health and Occupational Medicine (approval no. SDZFY-EC-A-2023-10).

### 2.2 DBT preparation

*Astragalus mongholicus Bunge* [Fabaceae*; A. mongholicus Bunge*] (2023030325) and *A. sinensis (Oliv.) Diels* [Apiaceae*; A. sinensis (Oliv.) Diels*] (221250) were provided by the pharmacy of Shandong Provincial Hospital of Traditional Chinese Medicine (Jinan, China). According to the recommended human dose, the mass ratio of *A. sinensis* and *A. mongholicus bunge* was 1:5. To align with clinical medication practices, the two botanical drug mixtures were made into decoctions rather than lyophilized powders (Chinese Pharmacopeia Commission, 2020). The mixture of the two botanicals was soaked in 8 times volume/weight distilled water for 30 min and decocted for 1 h. Subsequently, the liquid was poured off, and the remaining mixture was subjected to a second decoction by adding 4 times the distilled water volume. The two decoctions were mixed and filtered. Finally, a rotary evaporator concentrated the DBT decoction to a crude drug content of 0.76, 0.38, and 0.19 g/mL DBT solution, respectively.

The recommended clinical dosage of DBT for adults is 36 g crude drug/day, and the dosage for 60 kg adults is 0.6 g/kg/day (Chinese Pharmacopeia Commission, 2020). Based on the dose conversion method for experimental animals in “*Experimental Methodology of Pharmacology (4th edition)*” (Wei et al., 2020), the DBT dose for rats (average body weight: 200 g) was approximately 3.8 g crude drug/kg using a body surface area conversion factor of 6.3 (Xu, et al., 2002). Accordingly, the low, medium, and high doses of DBT were 1.9, 3.8, and 7.6 g/kg/d, respectively, equivalent to 0.5, 1, and 2 times the recommended daily dose of DBT.

### 2.3 Experimental design

The experimental design is presented in the graphic abstract. A “28-day exposure modeling +42-day intervention” regimen was introduced. Due to the strictly regulation on the bacterial/virus experiments with animals in China, the rat model for lung inflammation and injuries was established using a biohazard-free method. Silica particles (#SLCH2693; Sigma–Aldrich, Shanghai, China) were finely ground for 2 h, autoclaved at 120°C, and then suspended in 0.9% saline at a concentration of 100 mg/mL. The rats were assigned to six groups (*n =* 10 each): (1) blank, (2) DBT, (3) silica, (4) silica + high-dose DBT, (5) silica + medium-dose DBT, and (6) silica + low-dose DBT. We injected 1 mL of sterile saline drops into the trachea of rats in groups 1 and 2, and groups 3–6 received 1 mL of the silica suspension. After 28 days, the rats in groups 1 and 3 received daily gavage with sterile saline, whereas the rats in group-2, -4, -5 and -6 received daily DBT doses of 7.6, 7.6, 3.8, and 1.9 g/kg body weight, respectively.

Per each experimental group, 5 rats were euthanized at day-56 and the other 5 rats were euthanized at day-70 (see *Graphic Abstract*). Upon the finish of day-56 and day-70 (intervention completed), 5 rats per group were weighed and euthanized at each time point. Lung coefficients [lung weight (mg)/body weight (g)] were calculated. One-third of each left lung was fixed for histopathological and immunohistochemical (IHC) analysis. The remaining two-thirds of the lung tissue was homogenized thrice (30 s at 15 s intervals) in physiological saline at 4°C, using a polymer homogenizer, and the supernatant was centrifuged at 3,500 × *g* for 10 min. Homogenates capture both intracellular (e.g., TGF-β precursors) and extracellular (e.g., secreted cytokines) metabolites, providing a comprehensive representation of global lung pathology. The remaining lung tissue samples were divided and stored at −80°C.

### 2.4 Lung function evaluation

Lung function in the rats was evaluated on day-56 and day-70 (intervention completed) using a whole-body volumetric flow-through descriptive method (AAC060, EMMS WBP system; EMMS Technologies, Hampshire, Uinted Kingdom). Rats acclimatized to the testing environment 15 min before tidal volume (TV), frequency of breath (f), and pause enhancement (Penh) were recorded over a 10-min period to determine the baseline respiratory parameters, including those for airway obstruction.

### 2.5 Histopathology and immunohistochemistry

Sections (5 μm thick) were cut from paraffin-embedded lung blocks using a microtome (RM2245; Leica Microsystems, Wetzlar, Germany), and tissues were stained with hematoxylin and eosin (H&E) and Masson’s trichrome. Histopathological severity was quantified using ImageJ software (National Institute of Health, Bethesda, MD, United States) and validated via semi-quantitative scoring (Supplementary Tables 1, 2). For IHC, sections were treated with 1.0% potassium iodate and citrate buffer. Endogenous peroxidase was incubated for 10 min, washed with phosphate-buffered saline, blocked with normal goat serum, and incubated with the following antibodies overnight at 4°C: anti-nicotinamide adenine dinucleotide phosphate (NADPH) oxidase 4 (NOX4) (ab154244; Abcam, Cambridge, United Kingdom), anti-TGF-β1 (346599; ZEN-bioscience), anti-α-smooth muscle actin (α-SMA) (BM3902), and anti-Fibronectin 1 (FN1) (BM4460, Boster Bio, Wuhan, China). The next day, after returning to normal temperatures, the sections were flushed thrice with PBS, labeled with the secondary antibody, and incubated for 60 min at room temperature. Following another three PBS washes, streptomycin was applied for 60 min, followed by DAB color development. The stained sections were examined using an SP8 Leica laser confocal microscope (SP8, Leica).

### 2.6 Metabolomics, cheminformatics and bioinformatics

The metabolomic analysis of DBT metabolites was analyzed using Ultra High Performance Liquid Chromatography-Q exactive hybrid quadrupole orbitrap high-resolution accurate mass spectrometric (UHPLC-Q-Orbitrap HRMS, Thermo Fisher, United States). The liquid chromatography instrument is UltiMate 3000 UHPLC (Thermo Fisher Scientific, United States). The sample was eluted on a Thermo Hypersil gold C18 column (2.1 mm × 100 mm, 1.9 μm) with a gradient elution of 0.1% formic acid/water (A) and 0.1% formic acid/methanol (B) as the mobile phases, at a flow rate of 0.3 mL/min with an injection volume of 10 μL. The mass spectrometer was a Q-Exactive (Thermo Fisher Scientific, United States) equipped with a heated electrospray ionization (HESI) source, evaluated in positive and negative ion mode with a primary mass spectrometric (MS) scanning range of 100–1,500 m/z and a secondary MS scanning resolution of 17500. Data analysis was performed using Compound discover software (V 3.2, Thermo Fisher Scientific, United States).

The metabolites of *A. mongholicus bunge* and *A. sinensis* in DBT were acquired from the Traditional Chinese Medicine Systematic Pharmacology Database and Analytical Platform (TCMSP). OB (bioavailability) ≥ 30% and DL (drug-likeness) ≥ 0.18 were used as filtering conditions to identify the metabolites of DBT. Further screening was then performed using the Swiss ADME platform to select drugs with high gastrointestinal (GI) absorption. Then the retained metabolites were searched through Pubchem database. Only target genes shared by multiple metabolites (>2) were retained. Disease-related targets were ascertained in GeneCards and OMIM databases, and a score of ≥1.0 was used as the screening condition to obtain the corresponding disease targets.

Venny v2.1 was used to plot Wayne plots of target proteins of DBT metabolites versus pulmonary inflammation and fibrosis related proteins. In the String database, we imported the targets shared by the disease and DBT metabolites, selected “*Homo sapiens*” as the species, hid the free nodes in the protein network, and set the minimum interactions score to 0.9 to construct a visualized interactions network graph of the target proteins. Using the common target as the screening condition, the network relationship diagram of DBT–target was constructed using Cytoscape 3.10.2 by combining the relationships of diseases and targets of DBT obtained above. Gene ontology (GO) and Kyoto encyclopedia of genes and genomes (KEGG) enrichment analysis of 155 targets was performed using the cluster Profiler R package and visualized using SRplot.

### 2.7 Biochemical assays

Relative abundances of the following critical oxidative stress-related factors were measured in rat lung tissue homogenates using specific assay kits according to the manufacturers’ instructions: malondialdehyde (MDA, A003-1-2), superoxide dismutase (SOD, A001-3-2), glutathione peroxidase (GSH-Px, A005-1-2), and total antioxidant capacity (T-AOC, A015-2-1), all from Nanjing Jiancheng Biochemical Institute, Nanjing, China. Absorbances of the reaction solutions were measured at 532, 450, 412, and 405 nm, respectively, using an enzyme labeler.

### 2.8 ELISA

Cytokine levels in lung tissue homogenates from all groups were measured using the following ELISA kits: Tumor necrosis factor (TNF)-α (E-EL-R2856), Interleukin (IL)-6 (E-EL-R0015), IL-1β (E-EL-R0012), TGF-β1 (E-EL-0162), and IL-10 (E-EL-R0016), all from Elabscience, Houston, TX, United States, and p-SMAD3 (CB12063-Ra, Coibo Bio). Absorbances were recorded at 450 nm on an enzyme labeler (Sunrise, Tecan, Austria).

### 2.9 Western blotting

Lung tissue samples (100 mg) were processed by weighing and homogenizing for 30 min in freezing lysis buffer (P0013B, Beyotime Biotechnology, Jiangsu, China) containing the protease inhibitor phenylmethylsulfonyl fluoride (PMSF; Amresco 0754, Biosharp, Tallin, Estonia). Prior to use, the sample buffer was mixed with a ×5 master mix, and the supernatant of the lung tissue homogenates was boiled in water for 5 min. The plate was removed, and the samples were added (SM-W008-1, Protein Simple, San Jose, CA, United States) and incubated with the following antibodies: anti-NOX4 (1:100), anti-TGF-β1 (1:100), anti-α-SMA (1:50), anti-FN1 (1:100), and anti-β-actin (1:100). The plate was then loaded into a fully automated quantitative protein expression analysis system (Wes, Protein Simple) to analyze the results.

### 2.10 Statistics

Data were processed using the IBM SPSS version 25.0. Results are presented as means ± standard deviation (SD), and comparisons between groups were performed using tukey’s honest significant difference or Dunnett T3 tests. Histograms were plotted using GraphPad Prism 9.5.

## 3 Results

### 3.1 Metabolomic profile of DBT metabolites

A total of 159 features were identified in positive and negative ion mode by LC-MS to detect possible chemical metabolites in DBT, among which major metabolites, such as kumatakenin, formononetin, and calycosin, were detected. The relative percentage content of each major metabolite type was measured by area normalization. Among them, the trichothecene calycosin, formononetin, astragaloside IV, and ferulic acid contents reached 5.48%, 5.05%, 3.53%, and 3.35%, respectively. The detected metabolites are listed in Supplementary Figure 1.

### 3.2 Lung-protecting function evaluation of DBT

**FIGURE 1:**
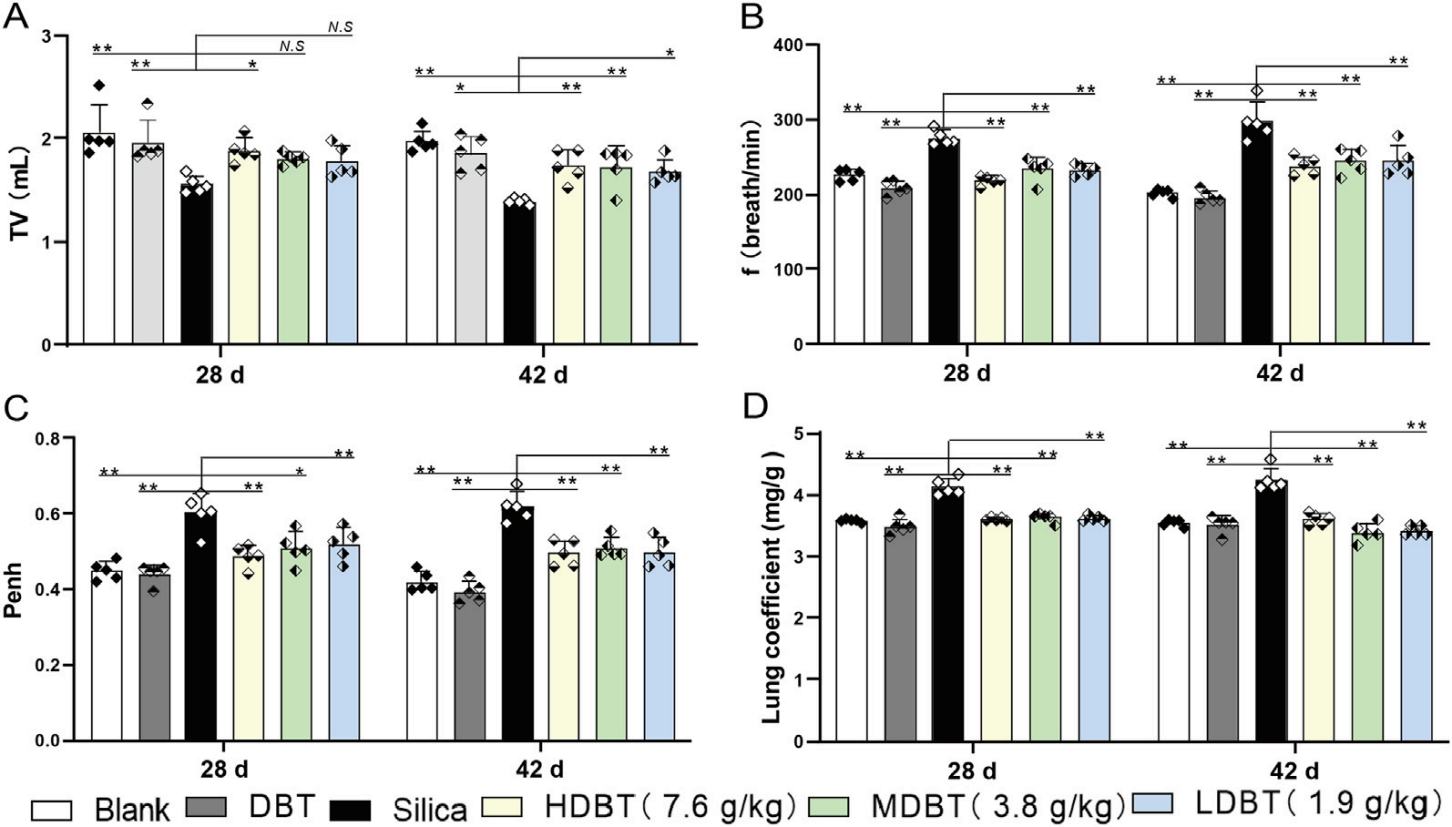
Effect of DBT on lung function and coefficients in rats. Determination of **(A)** tidal volume (TV), **(B)** frequency of breath (f), **(C)** pause enhancement (Penh), and **(D)** lung coefficient; *n* = 5, *P < 0.05 or **P < 0.01; *N.S.*, not statistically significant, compared with the silica group.

**FIGURE 2:**
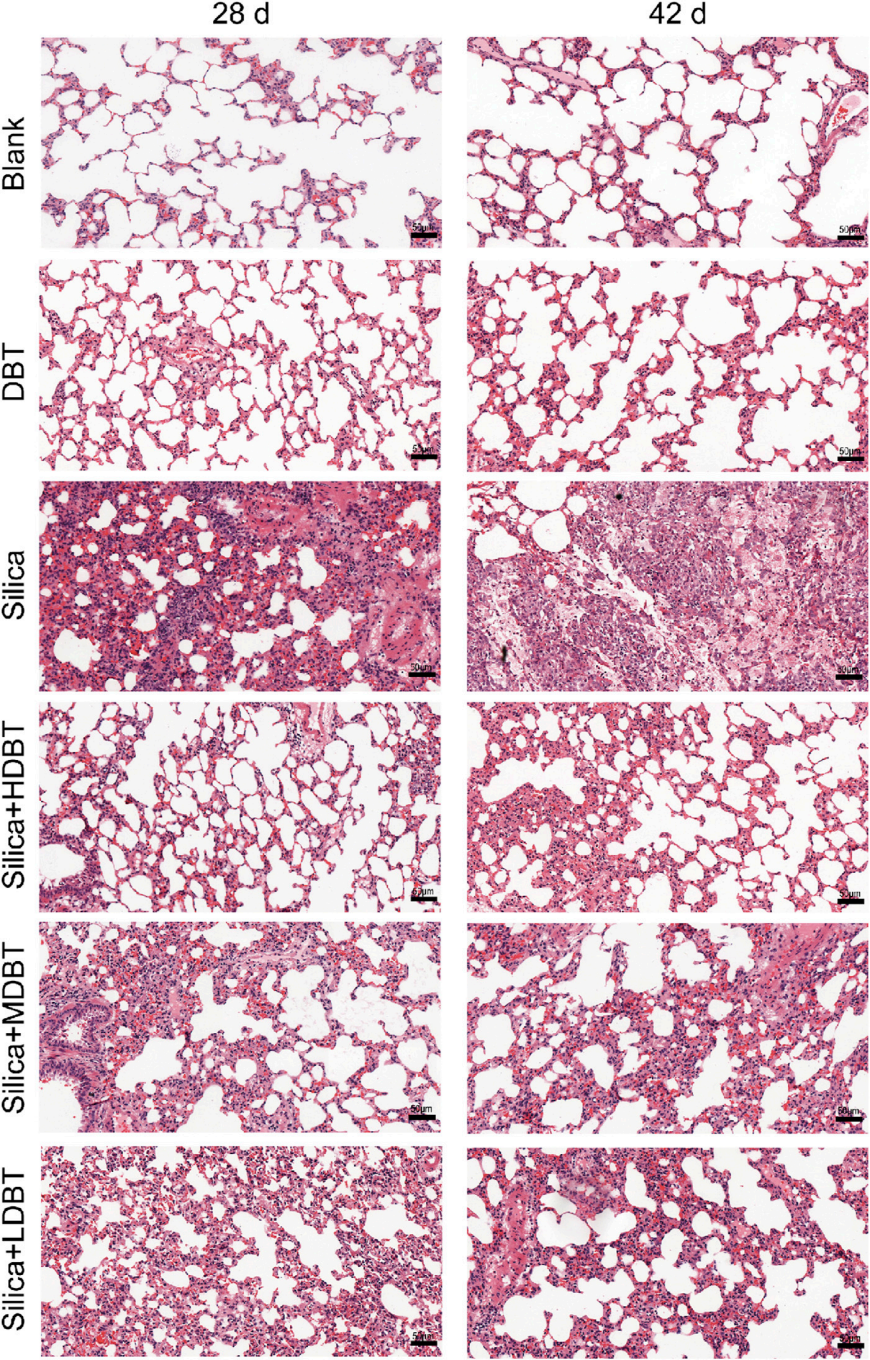
Hematoxylin and eosin (H&E) staining of lung tissue sections (magnification, ×200).

**FIGURE 3:**
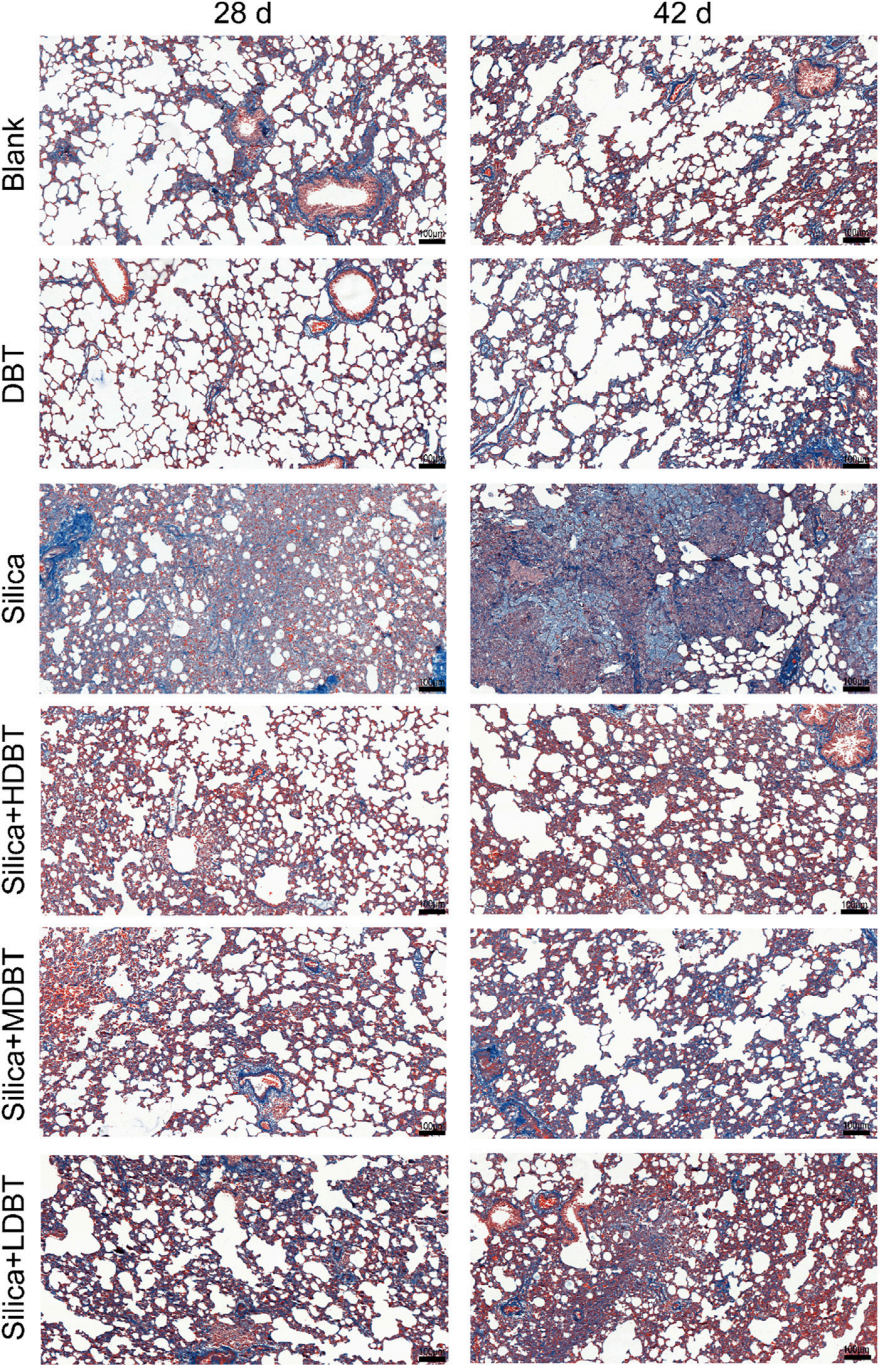
Masson’s trichrome staining of lung tissue sections (magnification, ×100).

Shown in Figure 1, lung function tests revealed that the lung function TV of rats in the silica group was significantly decreased (*P* < 0.01), and the frequency of breath and Penh were substantially higher (*P* < 0.01) than in those of the blank group. Conversely, TV dramatically increased (*P* < 0.05), whereas frequency of breath and Penh dramatically declined (*P* < 0.05) in the DBT intervention groups (7.6, 3.8, and 1.9 g/kg) after 28 and 42 days compared with those in the silica group (Figures 1A–C). Lung coefficients of rats in the silica group were markedly higher (*P* < 0.01) than those in the blank group at both time points, whereas intervention of 7.6, 3.8, and 1.9 g/kg DBT significantly reduced these coefficients (*P* < 0.01) (Figure 1D). H&E and Masson’s trichrome staining revealed structurally intact lung tissues and normal alveolar structures in the rats in the blank and DBT groups without inflammatory changes, whereas massive inflammatory cell infiltration and blue-stained collagen fibers, alongside characteristic silica nodules after prolonged exposure, were observed in the lung tissue of rats in the silica group. DBT intervention had a protective effect on silica-induced pulmonary fibrosis, reducing inflammatory cell infiltration and blue-stained collagen fiber deposition, and no silicosis nodules appeared in the lung tissues of the rats in the DBT intervention groups, with the most remarkable effect in the high-dose group. Semi-quantitative analysis confirmed significantly increased inflammation and collagen deposition in the Silica group compared to those in the Blank group (*P <* 0.01). However, these parameters were significantly attenuated with treatment with DBT on days 28 and 42 (*P <* 0.05; Figures 2, 3; Supplementary Tables 1, 2).

### 3.3 Functional pathway analysis of DBT

**FIGURE 4:**
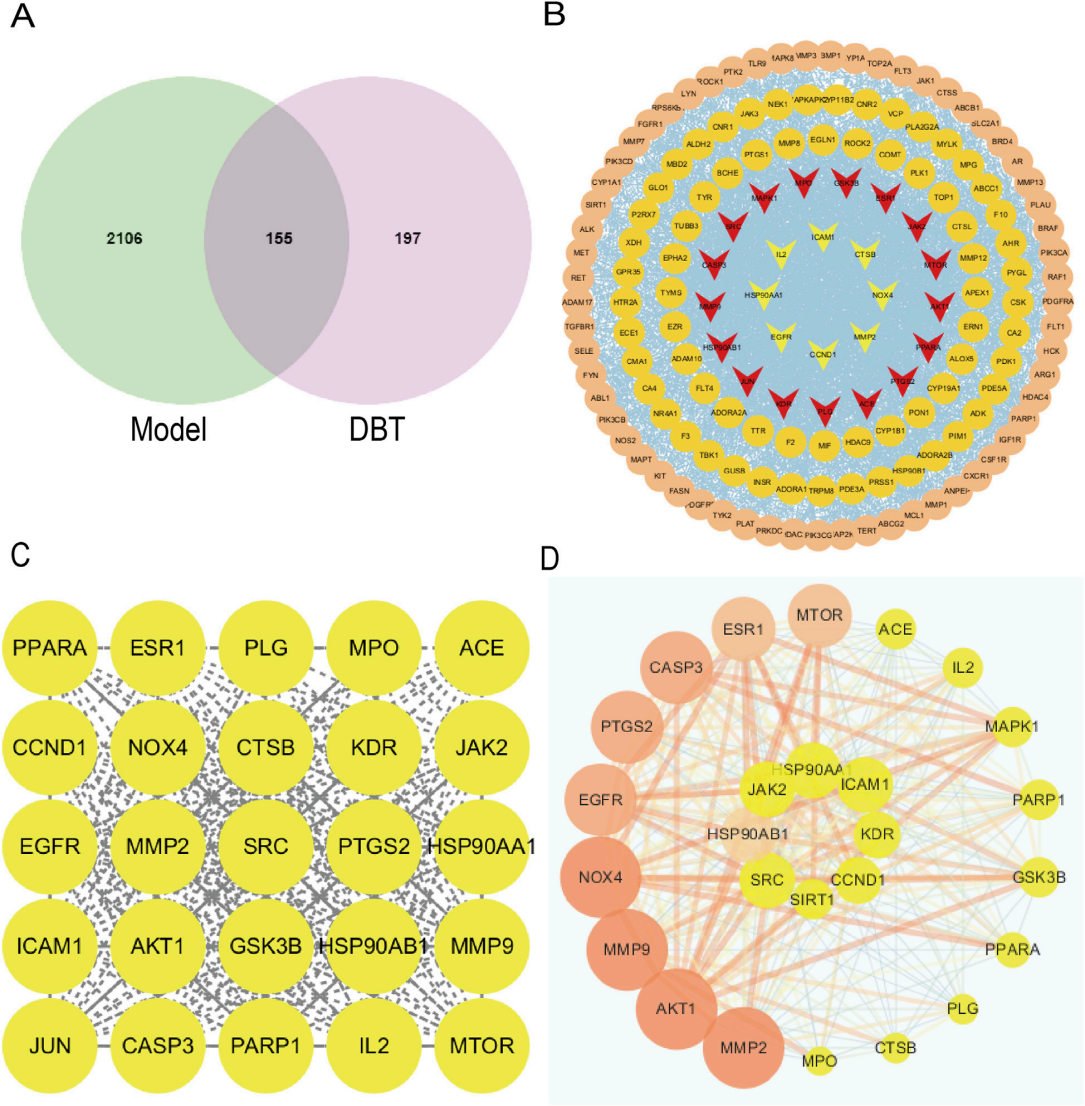
NOX4 as a common target of DBT and pulmonary fibrosis. **(A)** Wayne diagram of DBT and pulmonary fibrosis intersecting target. **(B)** PPI network of overlapping genes based on STRING. **(C)** DBT and pulmonary fibrosis common potential targets. **(D)** DBT and pulmonary fibrosis common core targets.

Shown in Figure 4, based on metabolomics data, a total of 22 metabolites of DBT were obtained through the TCMSP database. The target proteins corresponding to DBT in the TCMSP database were transformed through PubChem, and a total of 197 gene targets were obtained. A total of 2,106 disease targets were screened from the disease database, and the DBT-pulmonary fibrosis target Wayne diagram was drawn to obtain 155 common targets for DBT metabolites and pulmonary fibrosis. The 155 drug and disease cross-targets obtained were imported into the STRING database to construct a PPI network, and the network topology was analyzed using Cytoscape 3.10.2 software after removing the free nodes, which had a total of 155 points and 2,174 edges. Further filtering was performed based on the mean values of the network topology analysis parameters (Betweenness unDir ≥149.41, Closeness unDir ≥0.0033, Degree unDir ≥28.05). There was a total of 27 points and 274 edges in the resulting network, and after filtering based on the degree values, the top 10 targets were MMP9, MPO, HSP90AB1, SRC, MTOR, GSK3B, MMP2, NOX4, AKT1, and PPARA (Figure 4).

**FIGURE 5:**
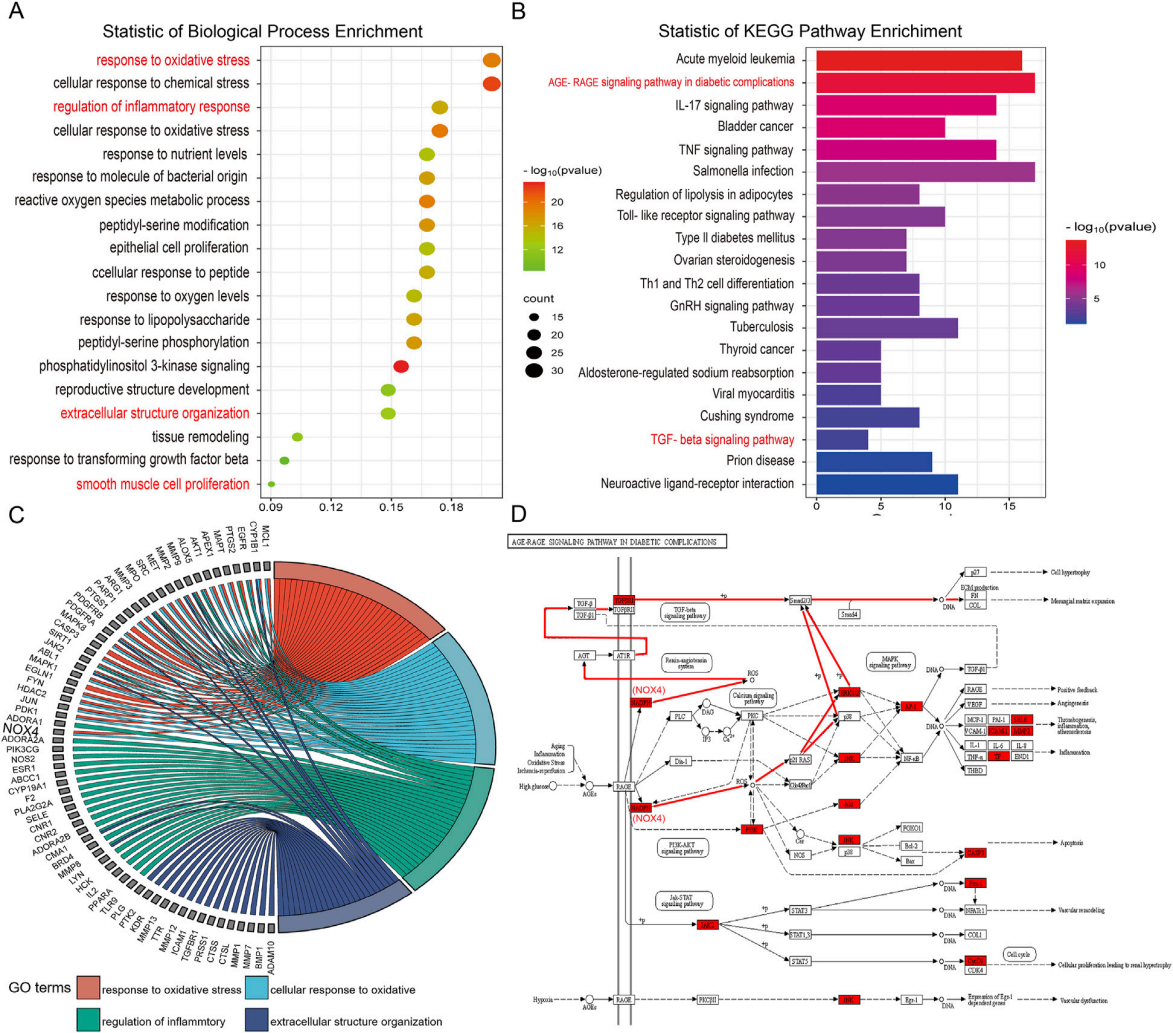
GO-BP and KEGG enrichment analysis. **(A)** GO biological processes enrichment analysis. **(B)** KEGG pathway enrichment analysis. **(C)** GO chord. **(D)** Schematic diagram of the AGE-RAGE signaling pathway.

Present in Figure 5, GO analysis of biological processes (BP) showed that genes involved in the regulation of pulmonary fibrosis by DBT were mainly enriched, potentially influencing inflammatory responses, oxidative stress, smooth muscle cell proliferation, and ECM deposition (Figure 5A). KEGG analysis showed that DBT-regulated pulmonary fibrosis genes were mainly enriched in the advanced glycosylation end product-receptor (AGE-RAGE), TNF, and TGF-β signaling pathways (Figure 5B). The AGE-RAGE signaling pathway produces protein glycosylation products (AGEs), induced by hyperglycemia, which bind to their receptors (RAGEs), in turn triggering a series of responses. The AGE-RAGE interaction involves four transduction signaling pathways: TGF-β, PI3K-AKT, MAPK-ERK, and NADPH oxidase-ROS.

Metabolite target analysis revealed that NOX4 is a key target of DBT in regulating lung fibrosis. The GO chord showed that NOX4 was highly associated with oxidative stress and inflammatory responses (Figure 5C), while AGE–RAGE signaling pathway mapping revealed that NOX4 drove the TGF-β signaling cascade response through reactive oxygen species (ROS) generation (Figure 5D).

Given the pivotal role of NOX4 in pathways and its strong correlation with the pathological features of pulmonary fibrosis, it was included as a core target for subsequent experiments. Based on the results of bioinformatics analysis and pilot experiments, we hypothesized that DBT ameliorated silica-induced pulmonary fibrosis by reducing NOX4 expression (Figure 5), attenuating oxidative stress and inflammatory responses, and reducing myofibroblast differentiation and ECM deposition through the TGF-β pathway.

### 3.4 DBT effects on NOX4, oxidative stress, and inflammatory responses

**FIGURE 6:**
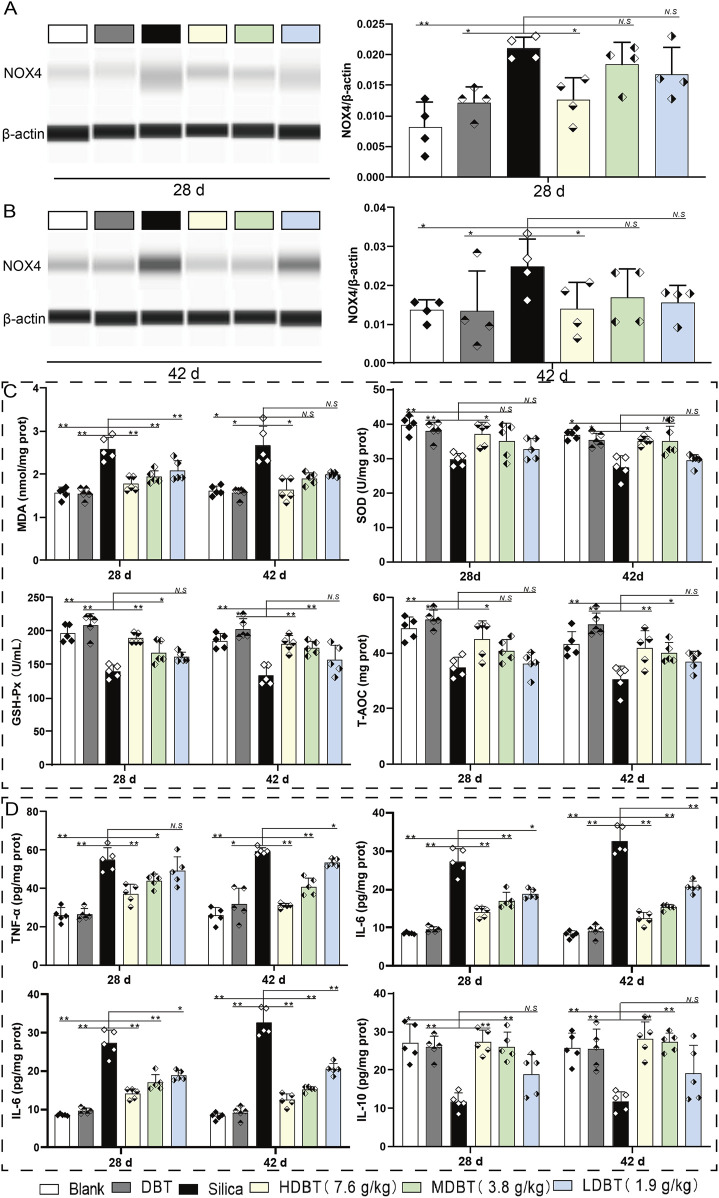
Effects of DBT on NOX4 expression and expression of oxidative stress indicators and inflammatory factors. NOX4 protein expression after **(A)** 28 and **(B)** 42 days of DBT administration, *n* = 4. **(C)** Levels of malondialdehyde (MDA), superoxide dismutase (SOD), glutathione peroxidase (GSH-Px), and total antioxidant capacity (T-AOC); **(D)** Levels of tumor necrosis factor (TNF)-α, interleukin (IL)-6, IL-1β, and IL-10; *n = 5* **(C, D)**, *P < 0.05 or **P < 0.01; *N.S.*, not statistically significant, compared with the silica group.

NOX4 protein expression was detected using Western blotting and IHC staining. NOX4 levels were significantly increased in rats (*P* < 0.05) following silica exposure compared with the blank group (Figure 6). However, 7.6 g/kg DBT intervention significantly decreased NOX4 abundance (*P* < 0.05) at both time points compared to the silica group (Figures 6A, B). IHC staining confirmed increased NOX4-positive staining in the lung tissues of rats following silica dust exposure. Different doses of DBT decreased NOX4-positive staining to various degrees (Supplementary Figure 2).

The oxidative stress index test showed that, compared with the blank group, the levels of the lipid peroxidation marker MDA were significantly increased (*P* < 0.05), whereas those of the antioxidants (SOD, GSH-Px, and T-AOC) were markedly decreased (*P <* 0.05) in silica-exposed lung tissues at both time points (Figure 6C). After 28 days of intervention, MDA levels markedly decreased in the 7.6, 3.8, and 1.9 g/kg DBT intervention groups (*P <* 0.01), with significant increases in GSH-Px in the 7.6 and 3.8 g/kg DBT intervention groups (*P <* 0.05) and SOD and T-AOC in the 7.6 g/kg group (*P* < 0.05), compared with the silica group. After 42 days of intervention, MDA levels were significantly reduced (*P <* 0.05) and SOD expression was significantly increased (*P <* 0.05) in the 7.6 g/kg DBT intervention group, while GSH-Px and T-AOC levels were significantly increased (*P <* 0.05) in the 7.6 and 3.8 g/kg DBT groups, compared with the silica group.

ELISA results showed that silica dust exposure markedly increased the expression of pro-inflammatory cytokines TNF-α, IL-6, and IL-1β (*P <* 0.01) while decreasing anti-inflammatory IL-10 abundance (*P <* 0.05; Figure 6D). After 28 days, TNF-α and IL-1β levels significantly decreased (*P <* 0.05), and IL-10 increased (*P <* 0.05) in the 7.6 and 3.8 g/kg DBT intervention groups compared with the silica group. Notably, IL-6 was significantly reduced in the 7.6, 3.8, and 1.9 g/kg DBT intervention groups (*P <* 0.05). After 42 days, TNF-α, IL-6, and IL-1β were markedly decreased in the 7.6, 3.8, and 1.9 g/kg DBT intervention groups (*P <* 0.05), whereas IL-10 was markedly increased in the 7.6 and 3.8 g/kg DBT intervention groups (*P <* 0.05) compared with the silica group.

### 3.5 DBT regulates TGF-β1–SMAD3 signaling

**FIGURE 7:**
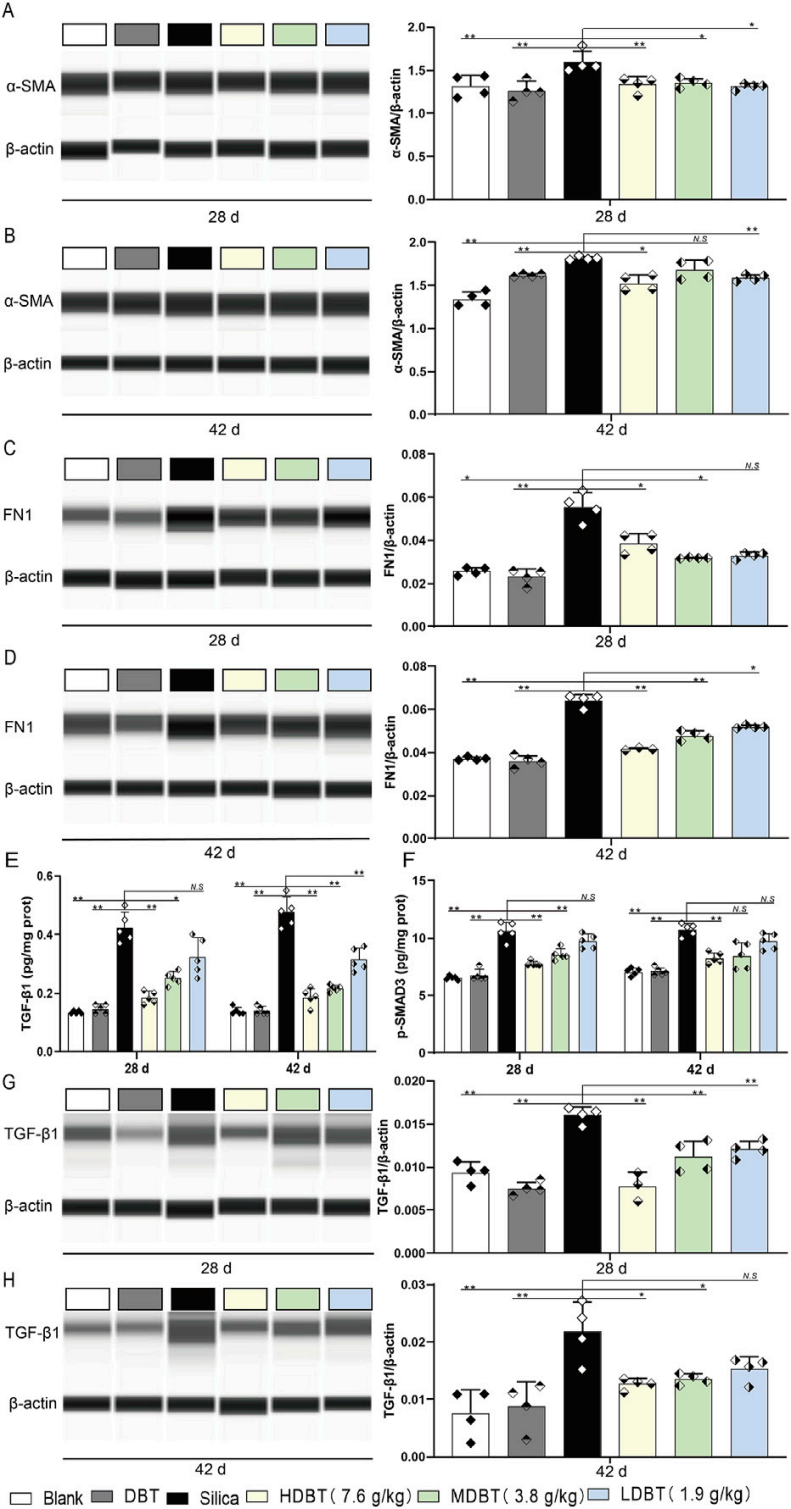
Effects of DBT on the expression of α-SMA, FN1, p-SMAD3 and TGF-β1 in rat lung tissue. α-SMA protein expression after **(A)** 28 and **(B)** 42 days of DBT administration. FN1 protein expression after **(C)** 28 and **(D)** 42 days of DBT administration; *n* = 4. **(E)** TGF-β1 and **(F)** p-SMAD3, *n* = 5. TGF-β1 protein expression after **(G)** 28 and **(H)** 42 days of DBT administration; *n* = 4 **(G, H)**, *P < 0.05 or **P < 0.01; *N.S.*, not statistically significant, compared with the silica group.

We assessed the expression of the myofibroblast differentiation marker α-SMA and ECM protein FN1 in lung tissue using Western blotting and IHC staining. α-SMA and FN1 levels were markedly increased in rat lung tissues after silica exposure at both time points (*P* < 0.01) compared with the blank group. After 28 days, α-SMA expression was significantly diminished in the 7.6, 3.8, and 1.9 g/kg DBT intervention groups (*P* < 0.05; Figures 7A, B). Similarly, FN1 expression was considerably reduced in the 7.6 and 3.8 g/kg DBT groups (*P* < 0.05) compared with the silica group. After 42 days, α-SMA was markedly diminished in the 7.6 and 1.9 g/kg DBT groups (*P* < 0.05), while FN1 was significantly reduced in all DBT intervention groups (*P* < 0.05; Figures 7C, D). Correspondingly, the IHC staining showed an increase in α-SMA and FN1 positive staining in rat lung tissues after exposure to silica dust, concentrated primarily in the ECM region. Administration of DBT reduced α-SMA and FN1 positive staining in the lung tissues of rats in the silica group (Supplementary Figures 3, 4).

TGF-β1 and p-SMAD3 levels were also markedly increased in the lung tissues of rats following silica exposure at both time points (*P* < 0.01) compared with the blank group. After 28 days of intervention, 7.6 and 3.8 g/kg DBT significantly decreased TGF-β1 and p-SMAD3 expression (*P* < 0.05) compared to the silica group. With prolonged intervention, 7.6, 3.8, and 1.9 g/kg DBT significantly diminished TGF-β1 expression (*P* < 0.05), whereas 7.6 g/kg DBT significantly reduced p-SMAD3 expression (*P* < 0.05; Figures 7E, F). Western blotting (Figures 7G, H) and IHC staining (Supplementary Figure 5) confirmed a significant reduction in TGF-β1 levels within the lung tissues of the silica group after DBT intervention at both time points.

## 4 Discussion

### 4.1 General mitigating effects

According to TCM, DBT is able to replenish qi and blood and improve the decline of internal organs due to qi and blood deficiency. Its pharmacological effects have been confirmed by *in vivo* and *in vitro* experiments, including improving hematopoietic function, promoting bone regeneration, and protecting the cardiovascular and pulmonary systems (Mak et al., 2006). We examined the chemical metabolites in DBT such as Ferulic acid, Calycosin, and Astragaloside IV by LC-MS, which is in agreement with the study of Kwan et al. (2019). Literature shows that Ferulic acid as a potential pharmacodynamic metabolite in the treatment of pulmonary fibrosis (Ali et al., 2021), Calycosin ameliorates acute lung injury by reducing inflammation (Zhu et al., 2021), Astragaloside IV ameliorates PM 2.5-induced lung injury and pulmonary fibrosis in rats (Tian et al., 2024).

Next, we established a lung inflammation and injury rat model using the non-exposed tracheal dust method to assess the effect of DBT on silica-induced pulmonary fibrosis and explore its potential mechanisms. Tracheal dripping of a silica suspension leads to alveolar injury, followed by a marked increase in neutrophils, macrophages, and lymphocytes, thereby inducing lung inflammatory exudates and progressive pulmonary fibrosis (Hu et al., 2014). After 28 days of tracheal drip silica suspension. We administered low, medium, and high doses of DBT for 28 and 42 days (shown in graphic abstract). Next, we examined lung function, lung coefficients and observed histopathological staining of rat lung tissues to explore whether DBT could ameliorate silica-induced pulmonary inflammation, early-fibrosis and tissue injuries in the rat model. The mechanism was explored based on metabolomics, bioinformatics and validation in animal samples through Western blotting and immunohistochemistry.

As mentioned in the introduction part, botanical drug decoction may provide an anti-biotic free and NSAID-free option for the children, elders and pregnant women to recover from lung inflammation, early-fibrosis and injuries. In this study we used a silica-induced lung inflammation model. It is a non-biohazard induced animal model because virus/bacterial treatment with experimental animals carries significant risk to public health. Lung function tests found that silica dosing groups had significant impairment of lung functions suggested by decreased TV and increased breathing frequency and Penh (Figure 1). Our function test shows that alveolar damage and decreased gas exchange function likely contributed to this impairment (Maher et al., 2023). Lung function impairment was also accompanied by increased lung coefficients, likely owing to a silica-induced inflammatory response that led to increased lung weight in the rats. The increase in lung weight and lung coefficient may also be caused by the elevation of collagen deposition, and stiffening of lung tissue in early fibrosis (Xie et al., 2023). Histopathological staining and semi-quantitative results revealed disorganized lung tissue in the silica group, characterized by thickened alveolar walls, likely due to inflammatory cell infiltration and increased collagen deposition (Figures 2, 3). However, DBT intervention attenuated these histopathological changes, improved lung function, and reduced lung coefficients at both time points. DBT alone did not affect lung function or coefficients, showing the safety of applied doses.

### 4.2 NOX4-TGF-β1-SMAD3 signaling and inflammatory responses and oxidative stress

NOX4 is a primary member of the NOX family predominantly expressed in lung endothelial cells. It is uniquely characterized as a constitutively active enzyme that produces hydrogen peroxide, which is tightly related to inflammatory responses and pulmonary fibrosis diseases (Qin et al., 2022). The specific mechanism of action of hydrogen peroxide within the rat model may include: (i) causing an oxidative/antioxidative imbalance and inducing oxidative stress injury and inflammation; (ii) elevating cytokine production by activating immune cells and promoting inflammatory responses (Barnes and Gorin, 2011); (iii) causing myofibroblast differentiation and ECM deposition by affecting the TGF-β1-SMAD3 signaling pathway (Amara et al., 2010).

NOX4 affected TGF-β1–SMAD3 pathway transduction (Figure 6), potentially through two mechanisms. Excess ROS generation can stimulate increased TGF-β1 expression, or ECM protein expression is upregulated via SMAD3 phosphorylation, regulating the myofibroblast phenotype in pulmonary fibrosis (Peng et al., 2019). A notable relationship exists between TGF-β1 and NOX4 during early lung fibrosis, with TGF-β1 increasing ROS levels in lung tissues by upregulating NOX4 expression (Wang et al., 2022). High ROS levels activate protein kinase C delta (PKC-δ), and phosphorylated SMAD3 mediates TGF-β1-induced myofibroblast activation (Jiang et al., 2014). NOX4 expression levels gradually increase with fibrosis progression, with the highest expression observed in advanced fibrotic tissues (Li et al., 2021). We observed increased NOX4 accumulation following exposure to silica powder, which progressively intensified with exposure time. Inhibiting NOX4 expression genetically or pharmacologically reduces ROS production, ameliorating BLM-induced pulmonary fibrosis in mice (Sato et al., 2016). This is consistent with our histopathologic findings. Additionally, we demonstrated that 7.6 g/kg DBT decreased NOX4 protein expression and oxidative stress. This was characterized by a decrease in the expression of MDA and an increase in the levels of the antioxidant indicators SOD, GSH-Px, and T-AOC.

Cytokines and growth factors induced by ROS can promote the inflammatory response (Wen et al., 2024) characteristic of pulmonary fibrosis. Under pathological conditions, inflammatory signals lead to the transformation of epithelial cells and fibroblasts to myofibroblasts (Gairola et al., 2024). In the current study, silica exposure upregulated the expression of inflammatory factors while downregulating anti-inflammatory mediators. DBT effectively suppressed the inflammatory response. Specifically, upon DBT administration, the pro-inflammatory factors TNF-α, IL-6, and IL-1β decreased, and the anti-inflammatory factor IL-10 increased.

### 4.3 TGF-β1-SMAD3 and early-fibrosis

DBT intervention also targeted early fibrosis. Injury to lung tissues triggers fibroblast activation, inflammatory mediator release, and ECM component production (e.g., collagen and fibronectin), initiating the healing response. However, severe or recurring damage can cause ECM metabolites to accumulate, leading to progressed tissue destruction and organ dysfunction (Henderson et al., 2020). In the current study, silica exposure increased TGF-β1 expression and SMAD3 phosphorylation and upregulated the myofibroblast marker α-SMA and ECM protein FN1 in rat lung tissues. The TGF-β1–SMAD3 signaling is a key regulator of DBT-induced antifibrotic effects (Meng et al., 2023). The pro-fibrotic effects of TGF-β1 are predominantly mediated via downstream SMAD3 phosphorylation (Zhang et al., 2024). Inhibiting the TGF-β1–SMAD signaling pathway by preventing TGF-β1 activation attenuates BLM-induced myofibroblast differentiation and ECM deposition (Hao et al., 2023). Accordingly, DBT treatment may protect against early fibrosis by inhibiting the TGF-β1–SMAD3 pathway and reducing myofibroblast differentiation and ECM deposition.

In summary, DBT significantly reduced NOX4 protein expression, improved oxidative stress, and regulated inflammatory responses. Hence, the antifibrotic effect of DBT might be achieved by reducing NOX4 expression to ameliorate oxidative stress and regulating the TGF-β1–SMAD3 pathway to ameliorate inflammatory responses fibrosis.

Given that this study sought to evaluate the effects of DBT on inflammation and fibrosis, it was administered via oral gavage for 28 days post-modeling, during which the inflammatory and fibrotic phases occur. Notably, inflammatory indicators did not differ significantly between 28 and 42 days of DBT administration. Given that the high inflammatory period generally occurs between 14 and 28 days (Li et al., 2024; Chanda et al., 2019), the lack of significant changes after this point may be attributed to the missed validation of the high inflammation period. Moreover, the expression of the fibrosis marker α-SMA did not significantly differ between the two time points, possibly due to the study being conducted during the fibrosis stage. Zhang et al. similarly reported no statistical difference in fibrosis-related marker expression in lung tissues of a silica-induced fibrotic rat model after 30 and 60 days (Zhang et al., 2019).

Gao et al., 2011 reported that DBTG ameliorates BLM-induced pulmonary fibrosis in rats by acting on TGF-β1 (Gao et al., 2011). Meanwhile, Wang et al., 2020 verified that a single dose of DBT inhibits BLM-induced lung fibrosis through the TGF-β1–SMAD3 pathway by inhibiting inflammatory factor secretion and collagen deposition (Wang et al., 2020). Zhao et al., 2015 observed that DBTG attenuates BLM-induced pulmonary fibrosis by reducing NOX4 expression and oxidative stress. However, they did not report time- or dose-dependent responses in α-SMA expression (Zhao et al., 2015). Notably, low-, medium, and high-dose groups were included in the current study, rather than a single dose. However, no significant dose-effect relationship was observed, which may be attributed to the multi-target nature of botanical drug medicines. The dose-dependent positive intervention effect suggests that appropriately increasing the DBT dose may enhance lung protective function. However, the results indicate that DBT may exert its effects as a whole rather than through individual metabolites.

Other studies have shown that decocting *A. sinensis* and *A. mongholicus bunge* at a 1:5 ratio results in more metabolites than simply mixing the two botanical drug extracts (Kwan, Dong and Tsim, 2021). Accordingly, we decocted these two species at a 1:5 mass ratio to prepare three DBT concentrations. A total of 159 metabolites were detected via LC-MS. Gao et al., 2011 and Zhao et al., 2015 used an 80% ethanol solution to extract and dry *A. sinensis* and *A. mongholicus bunge* and prepare three DBTG concentrations; the resulting decoctions were analyzed through fingerprinting, with average contents of 86% and 70.7%, respectively. Wang et al., 2020 prepared a single dose of DBT using a 1:5 mass ratio decoction but did not assess the metabolite content.

Although we established a silica-induced rat lung fibrosis model, others have employed BLM-induced lung fibrosis models (Gao et al., 2011; Wang et al., 2020; Zhao et al., 2015). Silica and BLM induce pulmonary fibrosis through different mechanisms. BLM triggers acute oxidative stress injury, while silica causes progressive fibrosis associated with physical stimuli, triggering a persistent inflammation–oxidative stress–fibrosis cascade. Notably, TCM functions through multiple targets and metabolites, and differences in modeling methods may lead to variations in DBT targets. Gao et al., 2011 and Wang et al., 2020 found that DBT exerts its antifibrotic effect by acting on TGF-β1. Meanwhile, similar to our results, Zhao et al., 2015 reported that DBTG reduces NOX4 expression by inhibiting oxidative stress.

Cumulatively, the findings of this study highlight NOX4 as a therapeutic target of DBT, suggesting its therapeutic potential in managing pulmonary fibrosis, particularly for patients unresponsive to conventional antifibrotics (e.g., pirfenidone). Moreover, the dual inhibition of NOX4 and TGF-β1–SMAD3 by DBT addresses oxidative stress and fibrotic signaling, offering a multitarget advantage over single-pathway drugs. Clinically, this finding could translate to reduced dosing frequency and minimized side effects. Metabolomic profiling of DBT further identified key metabolites, including calycosin and astragaloside IV, as potential quality control markers for standardizing DBT preparations. Future studies should incorporate parallel positive controls, such as pirfenidone, to evaluate the efficacy of DBT and explore synergistic interactions with conventional therapies.

## 5 Conclusion

In conclusion, our findings indicate that DBT attenuates pulmonary inflammation and early-fibrosis in rats. Through metabolomics and functional pathway analysis and *in vivo* experiments. We demonstrate that DBT exerts beneficial function through regulation on lung NOX4 expression. The mechanism may be related to the inhibition of oxidative stress, inflammatory response, and reduction of myofibroblast differentiation, and ECM deposition which is related to TGF-β1-SMAD3 signaling.

## Data Availability

The datasets in this article can be provided and made available upon request. All raw electrophoretic gels and blots pictures are included in the Supplementary Material.

## Abbreviations

α-SMA, α-smooth muscle actin; BLM, Bleomycin; DBT, Dang Gui Bu Xue Tang; ECM, Extracellular matrix; f, Frequency of breath; FN1, Fibronectin 1; GSH-Px, Glutathione peroxidase; H&E, Hematoxylin and eosin; IHC, Immunohistochemistry; IL-1β, Interleukin 1β; IL-6, Interleukin 6; IL-10, Interleukin 10; SOD, Superoxide dismutase; MDA, Malondialdehyde; NOX, NADPH oxidase; NOX4, NADPH oxidase 4; PMSF, Phenylmethylsulfonyl fluorid; PBS, Phosphate buffered saline; p-SMAD3, phosphorylated SMAD family member 3; Penh, Pause enhanced; ROS, Reactive oxygen species; TGF-β1, Transforming growth factor beta 1; TV, Tidal volume; TNF-α, Tumor necrosis factor α.
