# Supplementary material for: Astragalus mongholicus bunge and Angelica sinensis botanical drug decoction mitigates lung inflammation through NOX4/TGF-β1/SMAD3 signaling
Source: Front Pharmacol. 2025 Mar 26;16:1565569. doi: 10.3389/fphar.2025.1565569 (PMC11978833; doi:10.3389/fphar.2025.1565569)
Supplement: Supplementary file 1 [file DataSheet1.docx]

Supplementary Material

# Supplementary Figures


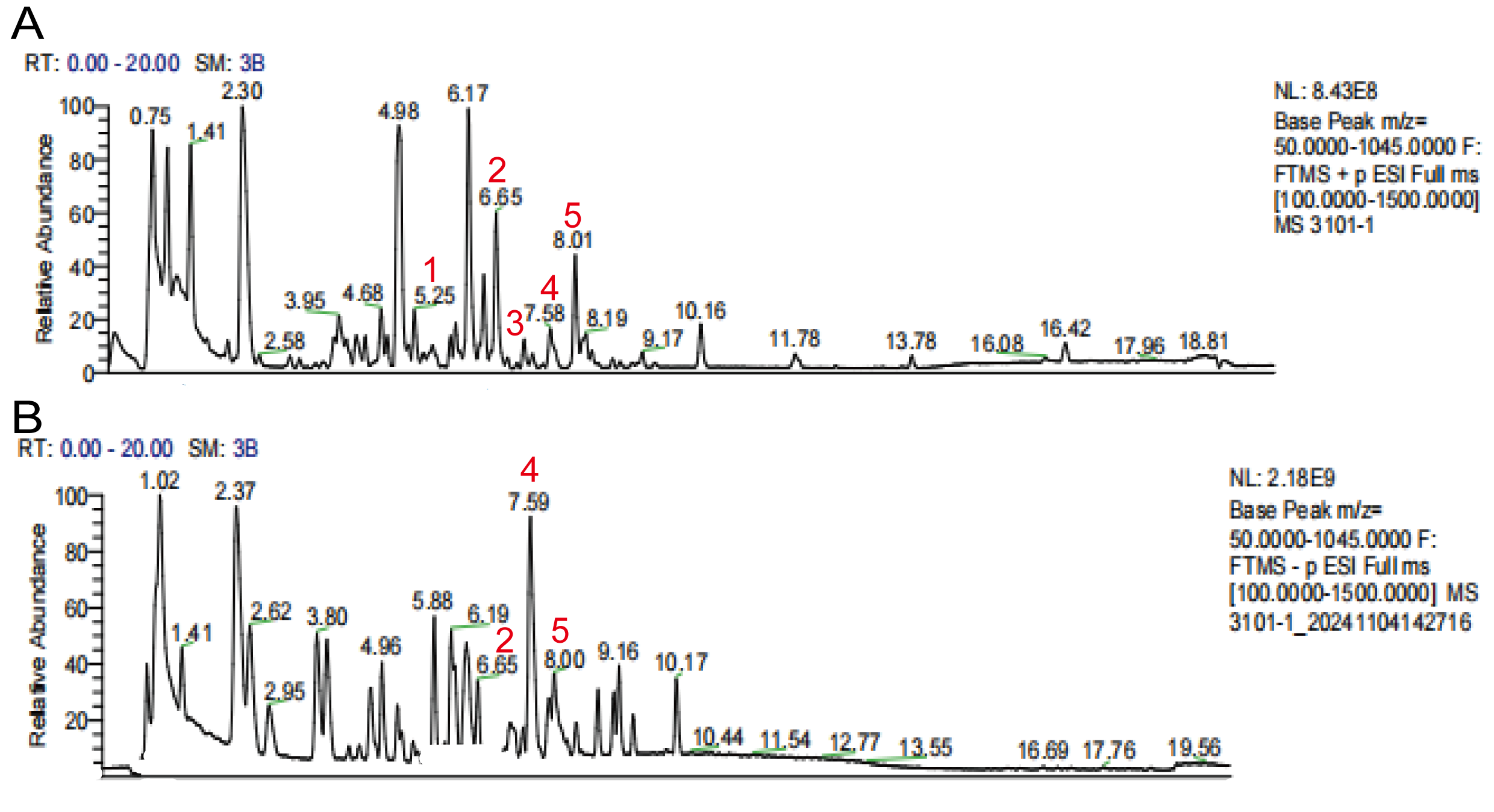


**Supplementary Figure 1.** LC/MS analysis of DBT. (A) Base peak chromatogram in positive ion mode; (B) Base peak chromatogram in negative ion mode. Identification of ferulic acid (1), calycosin (2), kumatakenin (3), astragaloside IV(4), and formononetin (5) was made by an MS detector.

**
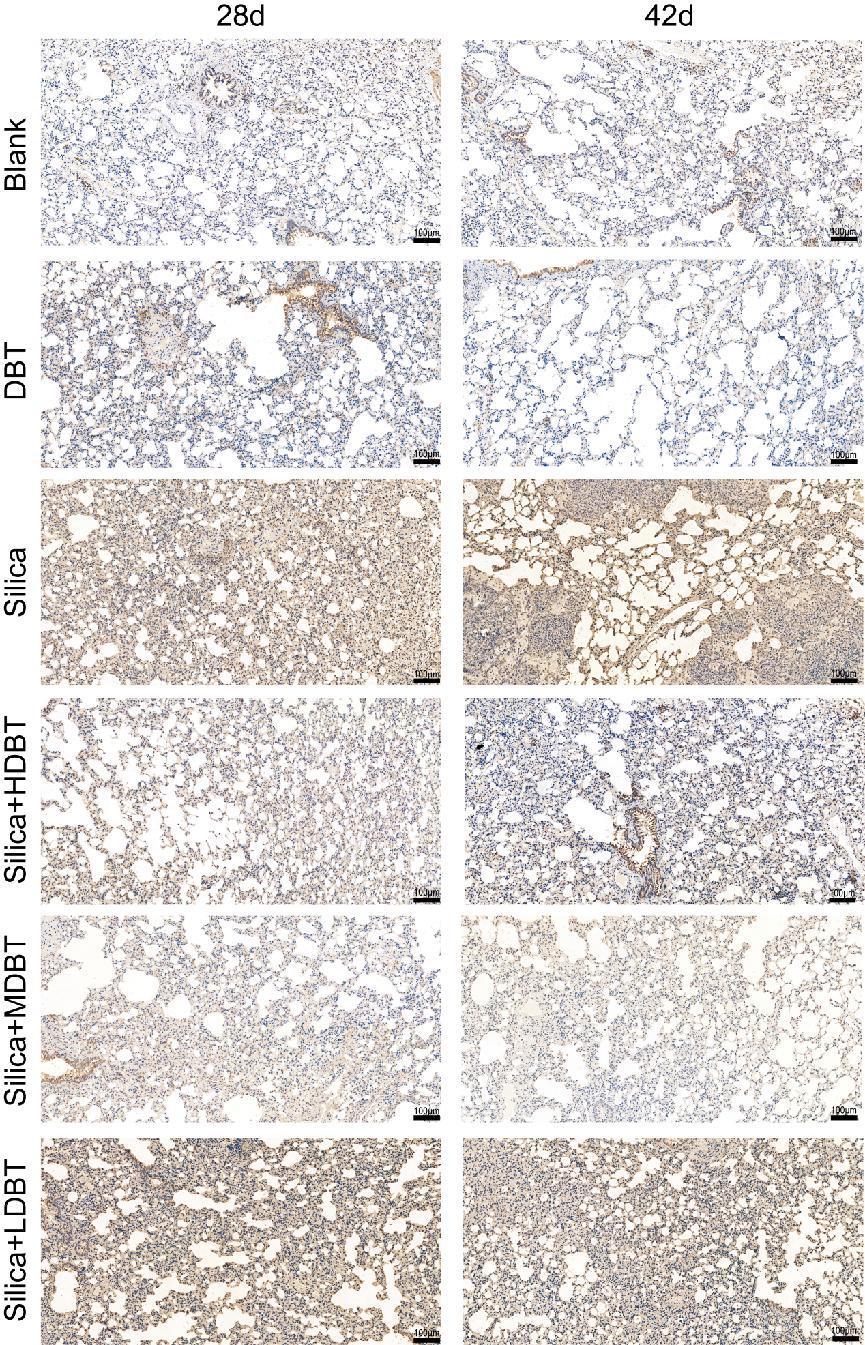
**

**Supplementary Figure 2.Immunohistochemical (IHC) analysis of NADPH oxidase 4 (NOX4) expression (magnification, 100×).**


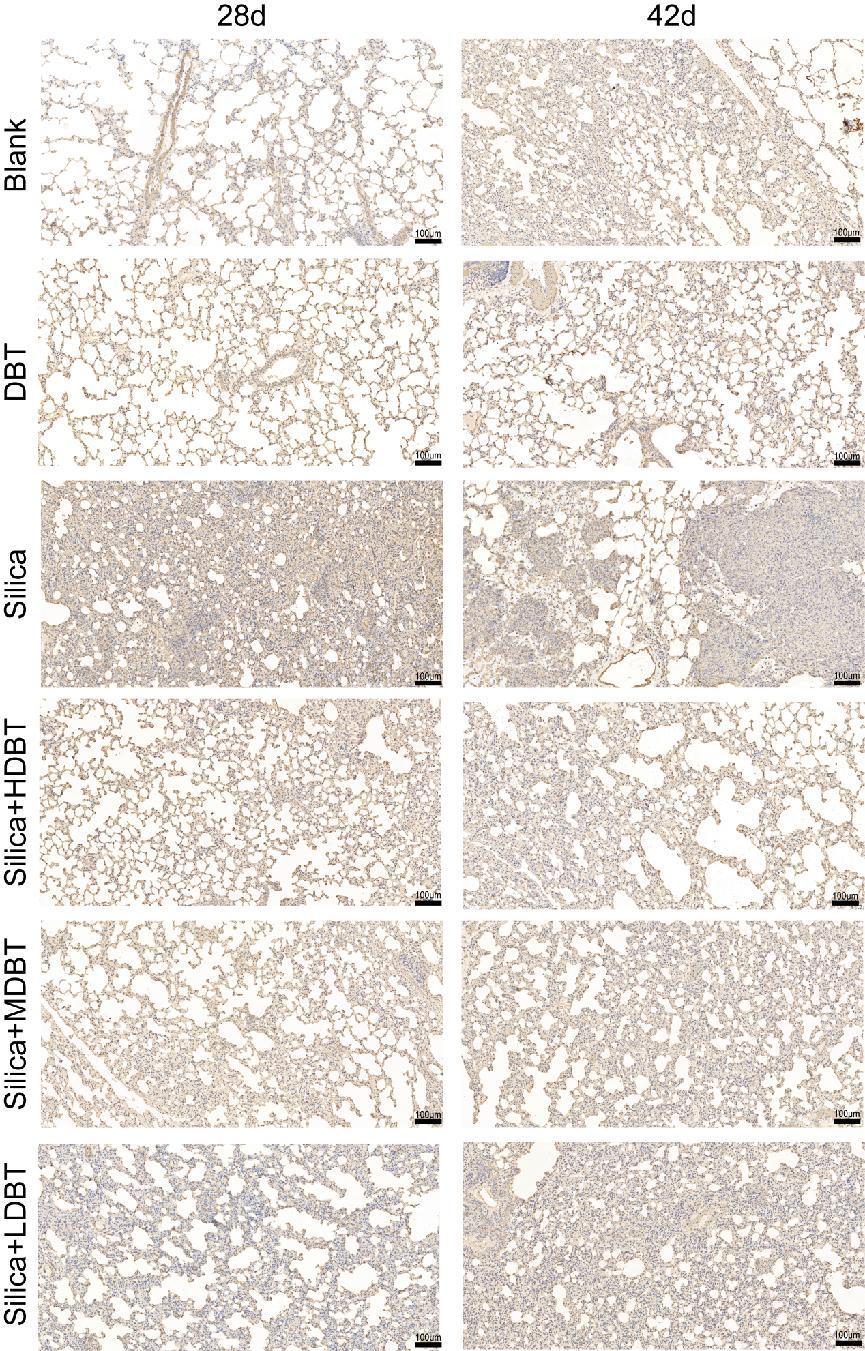


**Supplementary Figure 3. IHC analysis of α-smooth muscle actin (SMA) expression (magnification, 100×).**


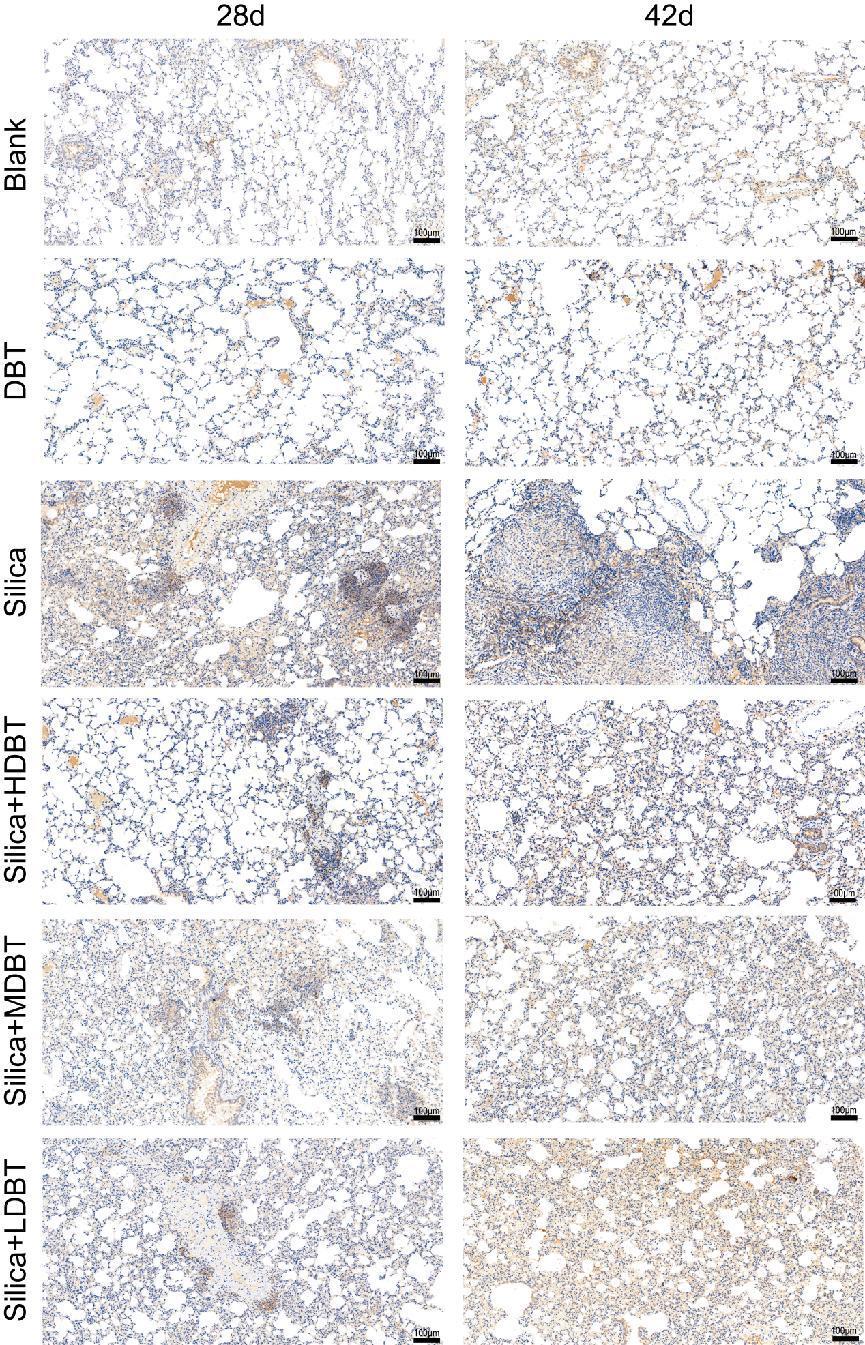


**Supplementary Figure 4. IHC analysis of fibronectin 1 (FN1) expression (magnification, 100×).**


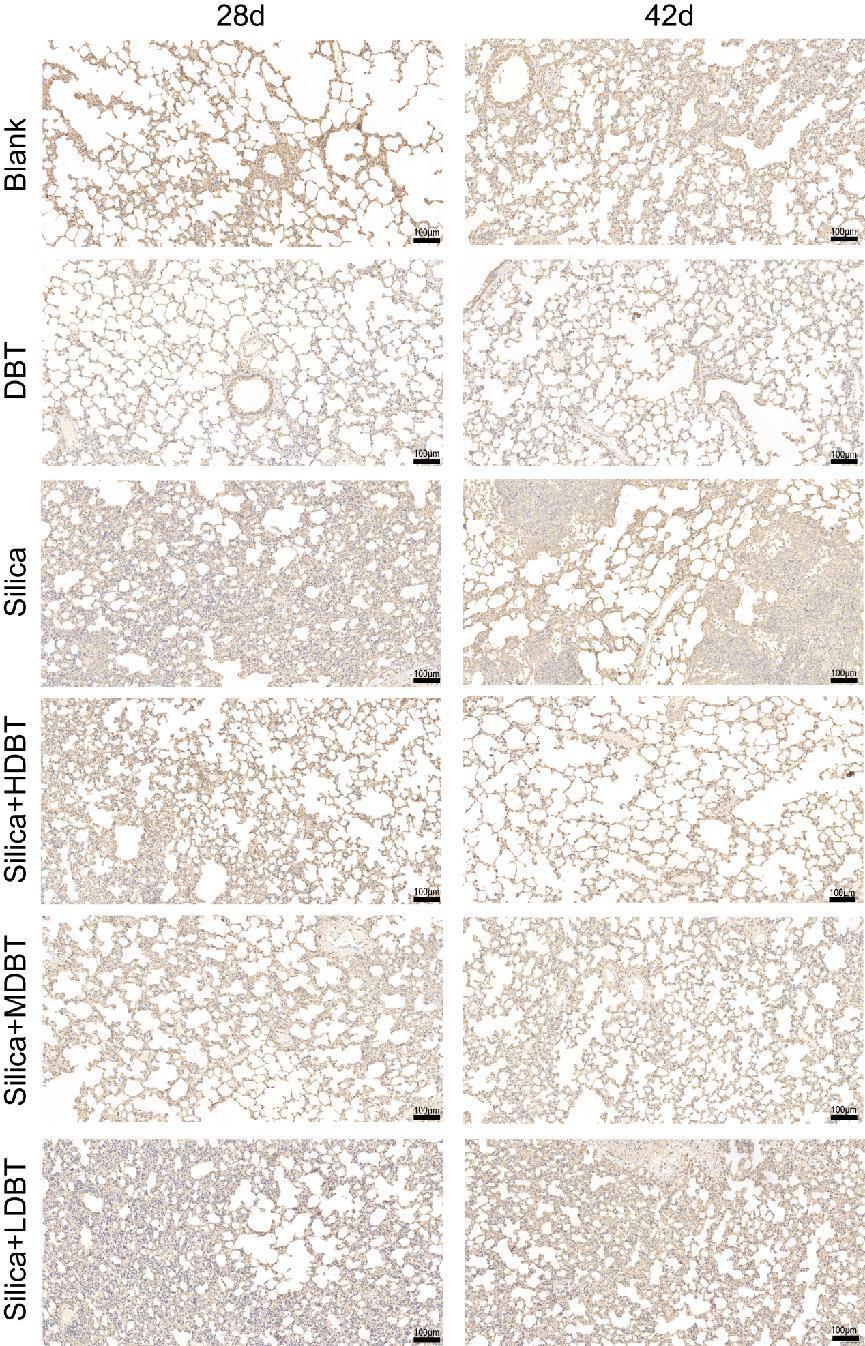


**Supplementary Figure 5. IHC analysis of transforming growth factor (TGF)-β1 expression (magnification, 100×).**

**Explanatory note:** The order of adding the samples was Blank group 1-4, DBT group 1-4, silica group 1-4, 1.9 g/kg DBT intervention group 1-4, 3.8 g/kg DBT intervention group 1-4, 7.6 g/kg DBT intervention group 1-4.


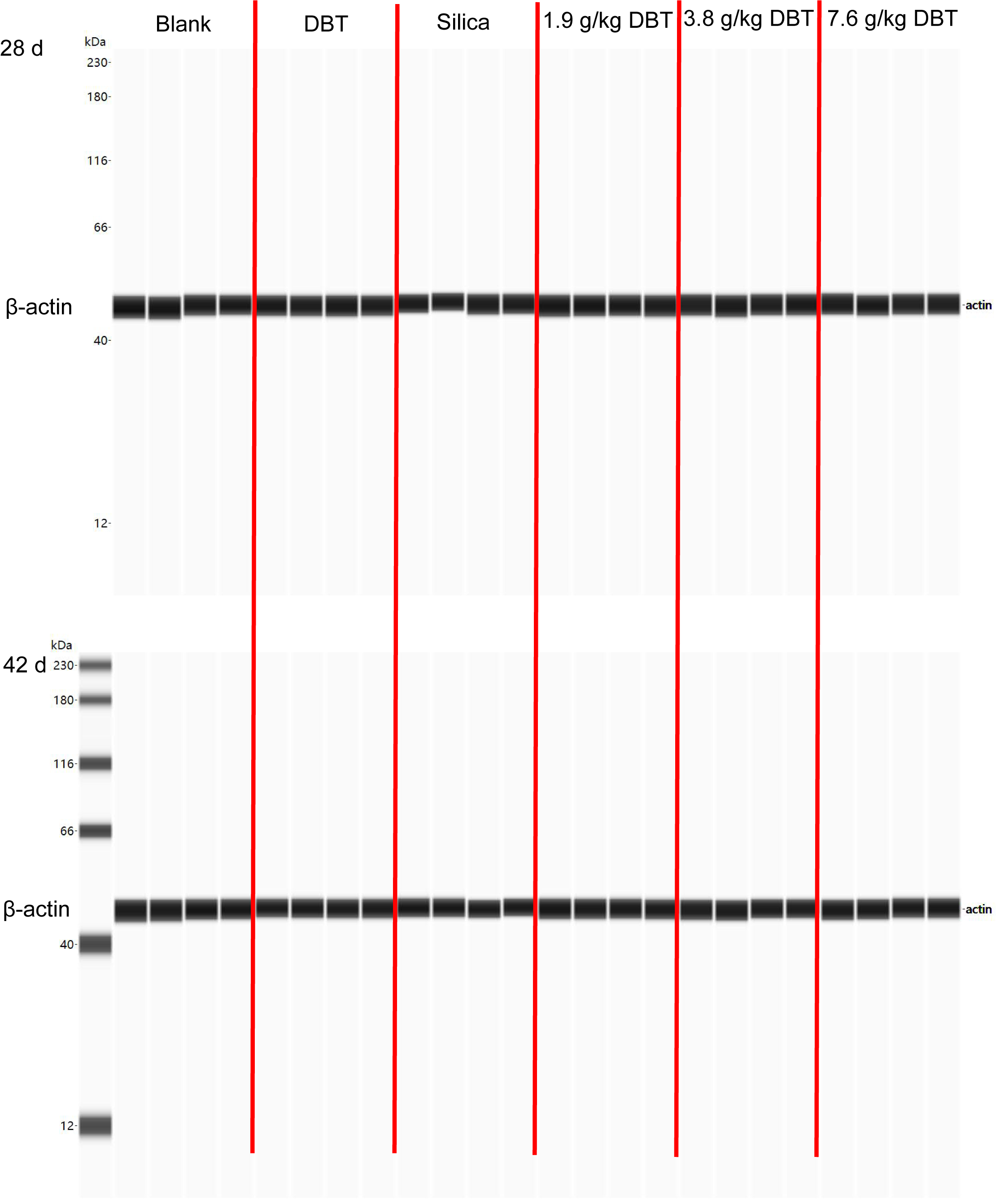


**Supplementary Figure 6.** Gel and blot images of β-actin protein


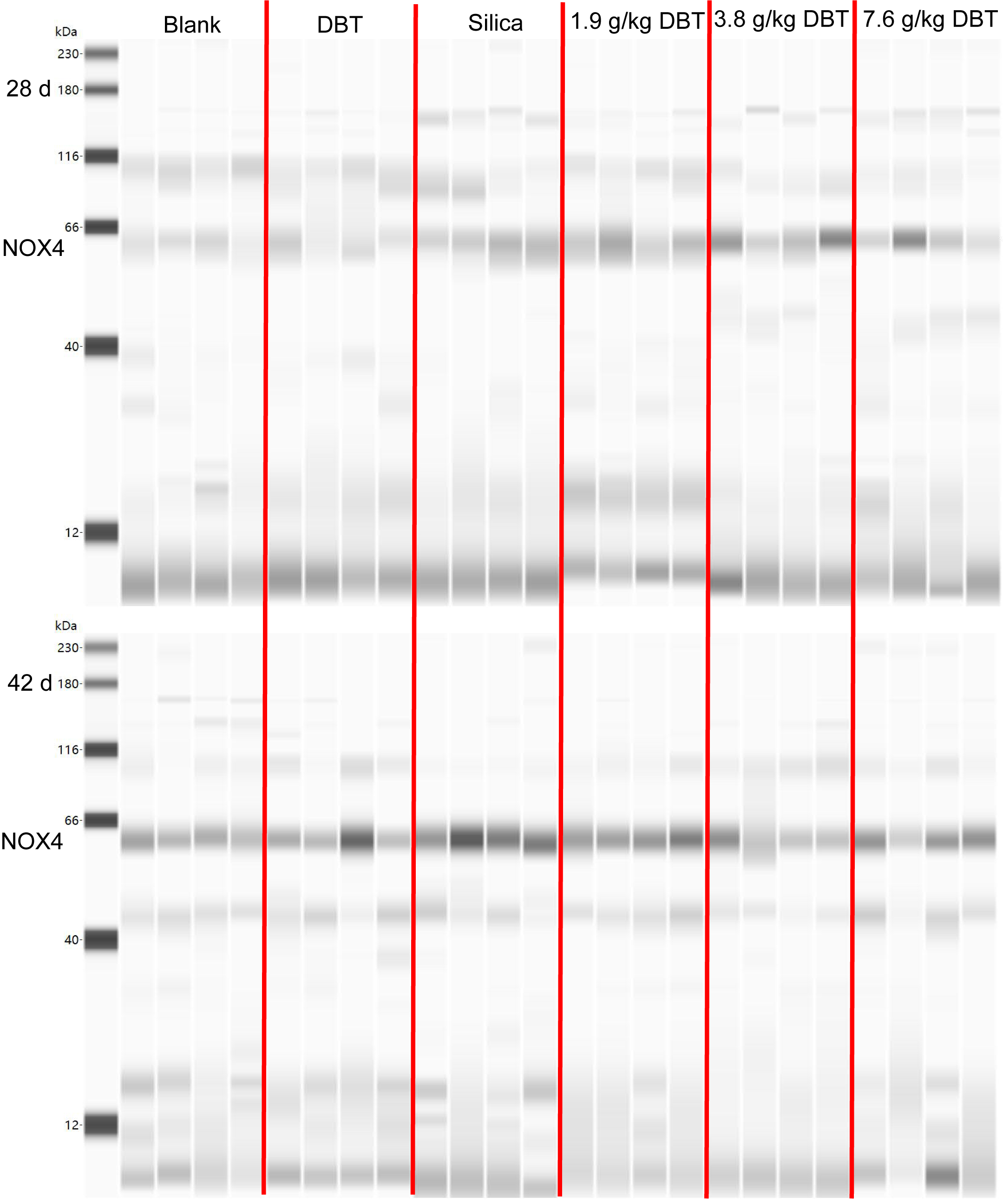


**Supplementary Figure 7.** Gel and blot images of NOX4 protein


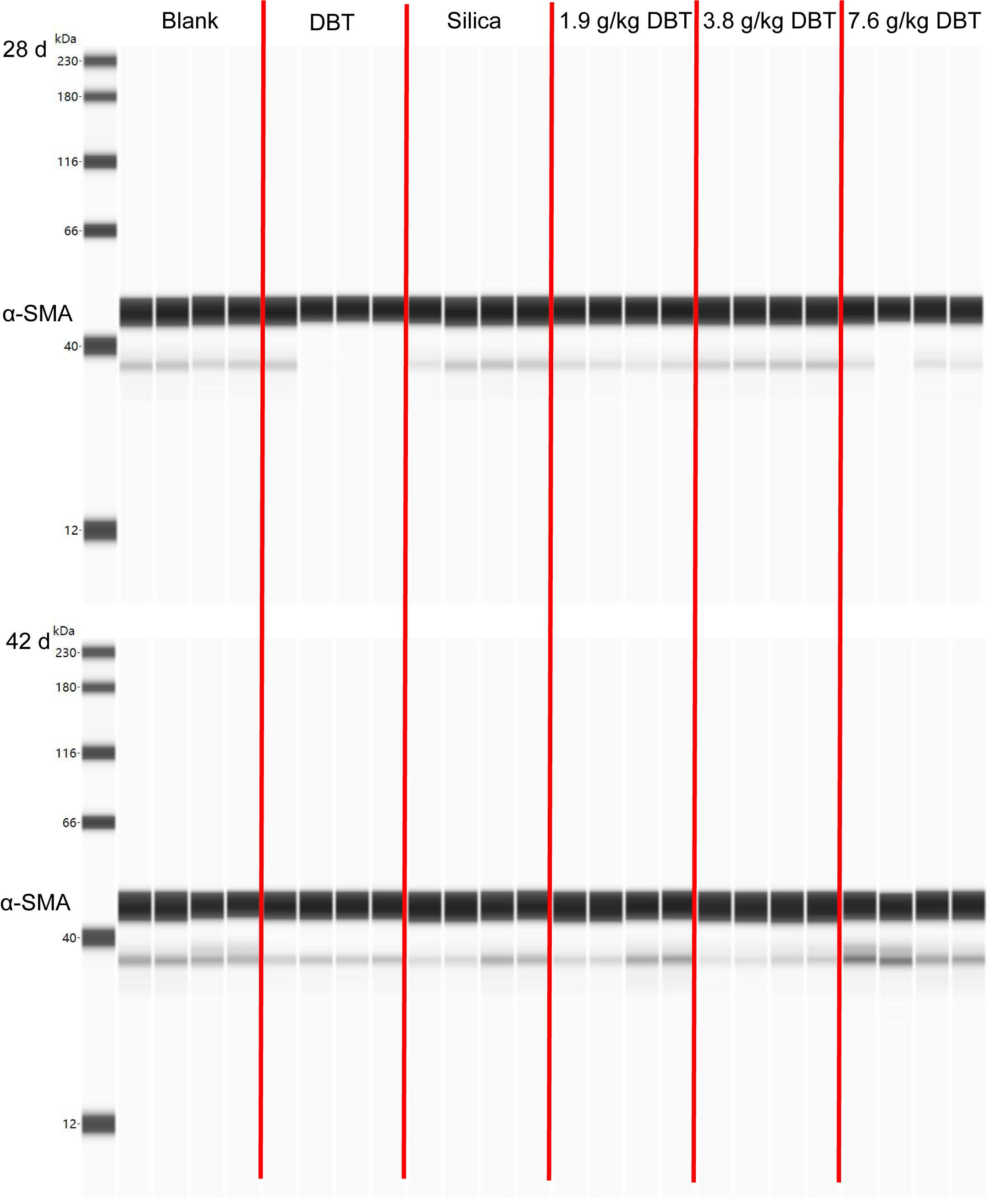


**Supplementary Figure 8.** Gel and blot images of α-SMA protein


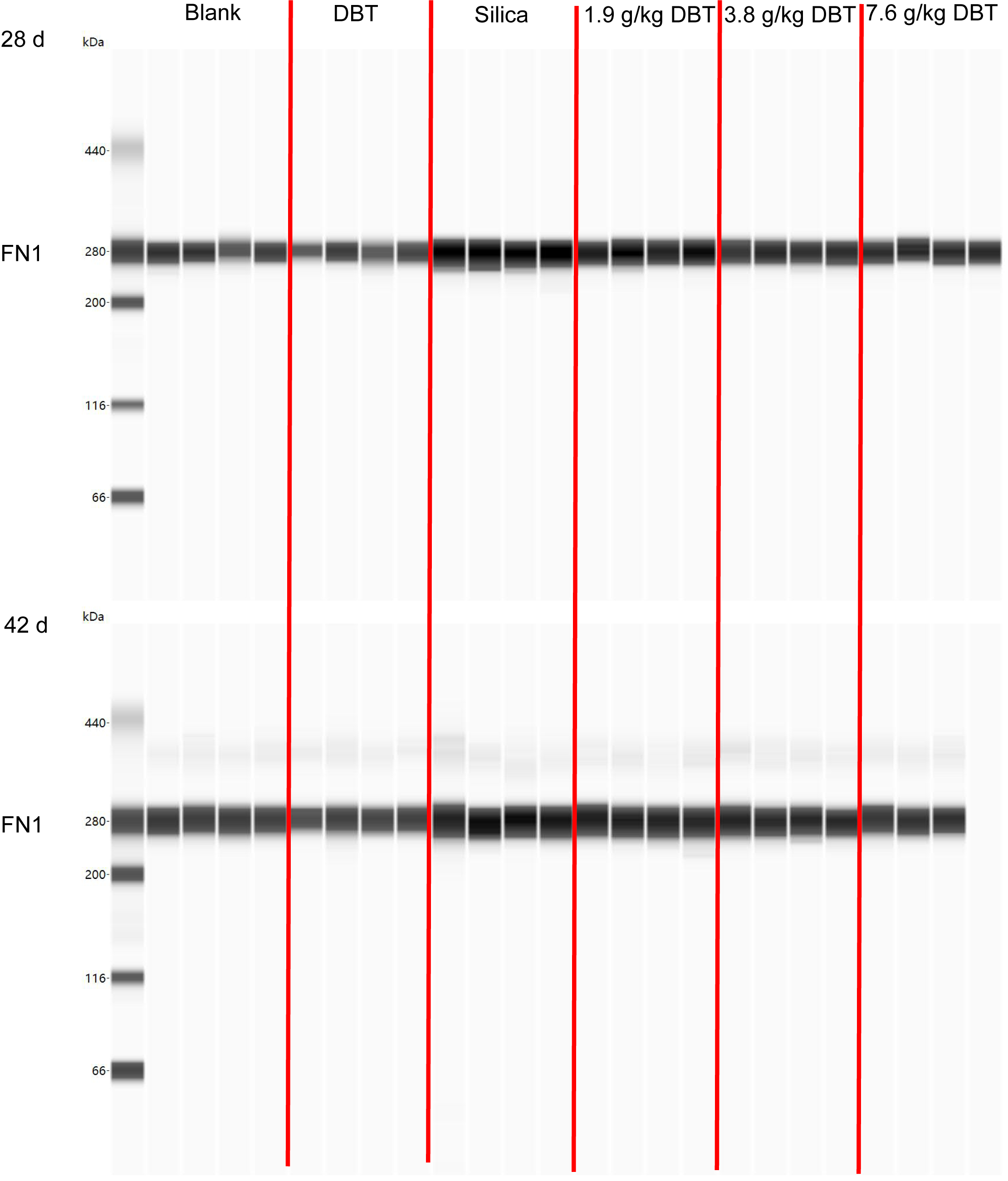


**Supplementary Figure 9.** Gel and blot images of FN1 protein


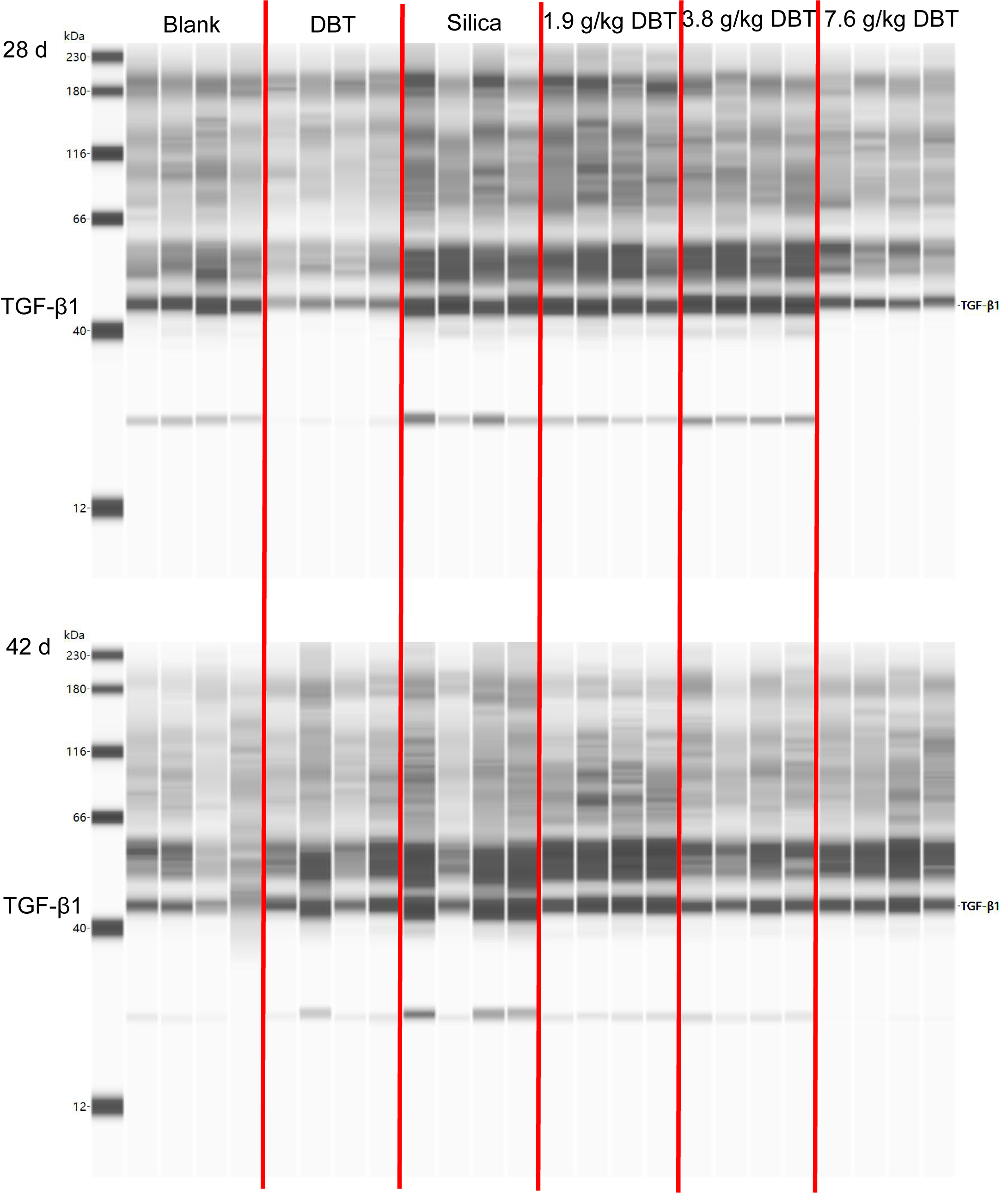


**Supplementary Figure 10.** Gel and blot images of TGF-β1 protein

**Supplementary Table 1. H&E semi-quantitative scale**

| Groups | Dose (g/kg•bw) | Inflammatory area/tissue area•100% | |
| --- | --- | --- | --- |
|  |  | 28 d | 42 d |
| Blank | / | 24.04±3.27** | 20.56±4.42** |
| DBT | 7.6 | 28.28±3.70** | 23.56±3.10** |
| Silica | / | 57.39±3.39 | 54.46±3.70 |
| HDBT | 7.6 | 45.70±3.16** | 41.55±2.75** |
| MDBT | 3.8 | 50.16±4.52* | 44.44±5.08** |
| LDBT | 1.9 | 54.05±3.55 | 52.41±3.09 |

Data are expressed as mean ± SEM (*n =5*), and one‐way ANOVA followed by Tukey’s multiple range test.

***P <* 0.01 and **P <* 0.05 as compared with Silica group.

**Supplementary Table 2. Masson’s trichrome semi-quantitative scale**

| Groups | Dose (g/kg•bw) | Collagen area/tissue area•100% | |
| --- | --- | --- | --- |
|  |  | 28 d | 42 d |
| Blank | / | 24.71±3.03** | 26.39±2.63** |
| DBT | 7.6 | 27.75±2.85** | 28.65±2.02** |
| Silica | / | 50.76±3.91 | 63.76±6.00 |
| HDBT | 7.6 | 41.68±3.26** | 40.84±4.38** |
| MDBT | 3.8 | 46.86±2.17 | 46.13±3.24** |
| LDBT | 1.9 | 47.72±5.55 | 49.20±5.12** |

Data are expressed as mean ± SEM (*n =5*), and one‐way ANOVA followed by Tukey’s multiple range test.

***P <* 0.01 and **P <* 0.05 as compared with Silica group.
